# A 2-decade bibliometric analysis of epigenetics of cardiovascular disease: from past to present

**DOI:** 10.1186/s13148-023-01603-9

**Published:** 2023-11-25

**Authors:** Yukang Mao, Kun Zhao, Nannan Chen, Qiangqiang Fu, Yimeng Zhou, Chuiyu Kong, Peng Li, Chuanxi Yang

**Affiliations:** 1 Department of Cardiology, The Affiliated Suzhou Hospital of Nanjing Medical University, Suzhou Municipal Hospital, Gusu School, Nanjing Medical University, Suzhou, 215000 Jiangsu China; 2 Department of Cardiology, The First Affiliated Hospital of Nanjing Medical University, No. 300 Guangzhou Road, Nanjing, 210029 Jiangsu China; 3 Department of Cardiology, Yangpu Hospital, Tongji University School of Medicine, 450 Tengyue Road, Shanghai, 200090 China; 4 Department of General Practice, Clinical Research Center for General Practice, Yangpu Hospital, Tongji University School of Medicine, Shanghai, 200090 China; 5 Department of Cardio-Thoracic Surgery, Nanjing Drum Tower Hospital, Affiliated Hospital of Medical School, Nanjing University, Nanjing, China; 6 Institute of Cardiothoracic Vascular Disease, Nanjing University, Nanjing, China; 7 Key Laboratory of Targeted Intervention of Cardiovascular Disease, Collaborative Innovation Center for Cardiovascular Disease Translational Medicine, Nanjing Medical University, 300 Guangzhou Road, Nanjing, 210029 Jiangsu China

**Keywords:** Bibliometric study, Epigenetics, Cardiovascular disease, Visualization, Bibliometric, VOSviewer, CiteSpace

## Abstract

**Background:**

Cardiovascular disease (CVD) remains a major health killer worldwide, and the role of epigenetic regulation in CVD has been widely studied in recent decades. Herein, we perform a bibliometric study to decipher how research topics in this field have evolved during the past 2 decades.

**Results:**

Publications on epigenetics in CVD produced during the period 2000–2022 were retrieved from the Web of Science Core Collection (WoSCC). We utilized Bibliometrix to build a science map of the publications and applied VOSviewer and CiteSpace to assess co-authorship, co-citation, co-occurrence, and bibliographic coupling. In total, 27,762 publications were included for bibliometric analysis. The yearly amount of publications experienced exponential growth. The top 3 most influential countries were China, the United States, and Germany, while the most cited institutions were Nanjing Medical University, Harbin Medical University, and Shanghai Jiao Tong University. Four major research trends were identified: (a) epigenetic mechanisms of CVD; (b) epigenetics-based therapies for CVD; (c) epigenetic profiles of specific CVDs; and (d) epigenetic biomarkers for CVD diagnosis/prediction. The latest and most important research topics, including “nlrp3 inflammasome”, “myocardial injury”, and “reperfusion injury”, were determined by detecting citation bursts of co-occurring keywords. The most cited reference was a review of the current knowledge about how miRNAs recognize target genes and modulate their expression and function.

**Conclusions:**

The number and impact of global publications on epigenetics in CVD have expanded rapidly over time. Our findings may provide insights into the epigenetic basis of CVD pathogenesis, diagnosis, and treatment.

**Supplementary Information:**

The online version contains supplementary material available at 10.1186/s13148-023-01603-9.

## Introduction

With the rapid urbanization and industrialization in modern civilization, cardiovascular disease (CVD) has gradually emerged as a world-class health killer that poses an unprecedented challenge to the life expectancy and the quality of life of hundreds of thousands of people and imposes an immense socio-economic burden worldwide, especially in middle- and low-income countries [1]. CVD is defined as a composite of various disorders that primarily jeopardizes the heart and central/peripheral blood vessels and interferes with the normal structures and functions of these vital organs responsible for maintaining blood circulation throughout the body, including heart failure (HF), coronary heart disease (CHD), arrhythmic disorders, stroke, and so on. According to the latest report from the American Heart Association (AHA), the prevalence of CVD and its related hospitalization and mortality display continuously increasing trends across the entire world, with approximately 19 million deaths being attributed to CVD in 2020, which increased by 18.7% compared to 10 years ago [2]. As for the etiology of CVD, genetic susceptibility, environmental exposures, aging, elevated blood pressure, undesirable lifestyles (e.g., smoking, physical inactivity), nutritional status (e.g., high dietary fat and/or cholesterol intake), and metabolic disorders (e.g., diabetes, obesity) have been well known as independent risk factors for CVD and thus become the current focuses in the prevention and treatment of CVD [2].

Although genome-wide association studies (GWAS) have identified numerous CVD-related DNA loci [3], most of the other risk factors for CVD have been proven to influence the cardiovascular system through epigenetic regulation, which refers to reversible and heritable alternations in gene expression in response to different external conditions in the context of the same DNA sequence, and predominantly incorporates DNA methylation, chromatin remodeling, histone modifications, and non-coding RNAs (ncRNAs) (e.g., microRNAs (miRNAs), long ncRNAs (lncRNAs), circular RNAs (circRNAs)) expression, thus phenotypically affecting CVD occurrence and outcome [4, 5]. The production of epigenetic markers commonly appears in the early stage of CVD, endowing these molecules with the potential to serve as candidate biomarkers for predicting and detecting sub-clinical CVD [6]. More importantly, due to the reversibility of gene expression, therapeutic strategies centered on epigenetic modifications may represent novel and promising approaches for CVD treatment in the future [7].

As a novel mechanism underpinning CVD occurrence, the role of epigenetic regulation in this process has captured extensive attention over the years, particularly in unveiling different epigenetic factors that can promote or prevent the development of CVD, and more efforts are still ongoing to decipher their diagnostic and therapeutic values for CVD. To date, a great amount of pre-clinical and clinical publications on epigenetics in CVD have been produced by researchers worldwide; nevertheless, an overview of the bibliometric profiles of these publications remains poorly understood. Since the concept of bibliometrics was first introduced by Prof. Alan Pritchard in 1969, it was soon developed as a separate discipline, in which a series of bibliometric indexes, including the amount and impact of publications, contributions of countries, institutions or authors, cooperation between different countries, institutions or authors were assessed, and used to roughly depict the developing landscape of a study theme. Recently, with the advent of the information era, several analytical software tools—such as Bibliometrics, VOSviewer, and CiteSapce—were designed and widely applied in processing big data and visualizing bibliometric information, facilitating the accurate identification of the up-to-date research trends and hotspot topics in a field of interest and the prediction of the frontiers and future directions [8]. Herein, we provide a contemporary overview of the guidelines for designing and performing a bibliometric study to facilitate a better knowledge of bibliometric methodology [9]. In the present study, we explored the bibliometric characteristics of the articles that investigated the role of epigenetics in CVD to yield a comprehensive knowledge of the major research status and summarize the evolving trends and hotspots during the past 2 decades.

## Methods

### Data source and search strategy

The data source for the bibliometric analysis was the Web of Science Core Collection (WoSSC), which is considered one of the most widely used databases for bibliometric research, predominantly because it can provide a comprehensive overview of relevant information including publications, citations, authors, references, and keywords [10]. The scope of data retrieval was limited to the Science Citation Index-Expanded (SCIE) database, without any restriction on language. Our search query combined Medical Subject Headings (MeSH) terms and keywords of multiple epigenetic factors with “heart” or “*cardi*”. The inclusion criteria were listed as follows: (a) documents published between 1 January 2000 and 31 December 2022; (b) document types of “articles”, “reviews”, “editorial material”, and “early access”, thus excluding “meeting abstract”, “proceedings paper”, “book chapter”, and any other non-relevant categories from the results. The retrieval procedure was implemented on a single day to mitigate confounding bias resulting from daily database updates to the maximum extent (December 31, 2022).

Moreover, to guarantee the authenticity and reliability of the research data, two well-trained investigators independently accomplished the mission of data collection, and another colleague was invited to participate in the discussion only in the case where divergent opinions occurred and needed to be resolved. We ultimately obtained 27,762 documents following manual reference screening, downloaded full records, and cited references in plain text for further analyses. A detailed flow chart of the study procedures is illustrated in Additional file 1: Fig. S1.

### Data analysis and visualization

We applied Bibliometrix (version 4.1.3), VOSviewer (version 1.6.17), and CiteSpace (version 5.8.R3) to assess three major bibliometric characteristics: co-citation, co-occurrence, co-authorship, and bibliographic coupling. A co-citation network is built based on the frequencies of two references/authors/countries/institutions being cited together [11]. Co-occurrence refers to a situation wherein two keywords appear in the same papers. Co-authorship is commonly used to depict a circumstance in which two research entities equally or hierarchically contribute to a study; therefore, this index reflects the cooperation between different authors, countries, or institutions. Bibliographic coupling is created based on the theory that scientific papers sharing similarities in reference citations are likely to be topically related to each other, and is considered an index that can reflect the relatedness between previous publications [12]. To track the research trends that emerged more recently, we also reduced the study period to the last 5 years (2017–2022) and the last year (2022) and repeatedly performed all the analyses.

Bibliometrix is an R-based tool designed for constructing a comprehensive science map of the published literature and is freely accessible on GitHub (https://github.com/massimoaria/bibliometrix) [13]. Specifically, Bibliometrix was used in this study to generate a quantitative estimation of annual publication outputs and major journals and to predict future research trends. VOSviewer enables graphically depicting and analyzing bibliometric indexes (https://www.vosviewer.com) [14]. Herein, we applied VOSviewer for implementing bibliographic coupling analyses of different countries, institutions, journals, authors, and references and networks of co-citation, co-authorship, and co-occurrence of keywords, in which each node corresponds to an individual object, with the size of the node and the thickness of the line connecting two nodes being proportional to the amount or frequency and the strength of the cooperative/co-cited/co-occurring associations between different objects, respectively. In addition, clusters that share similarities in particular attributes were marked with the same color in the network.

CiteSpace is a Java-based software tool designed by Prof. Chaomei Chen and is widely used in visualizing bibliometric features and predicting evolving trends of a research field [15]. In this study, we utilized CiteSpace to perform clustering, timeline, and burst analysis of co-cited references and co-occurring keywords and build co-citation and co-authorship networks. All cluster labels were extracted from the keywords based on the log-likelihood test (*P* < 0.001) and carefully re-checked to determine whether necessary modifications were needed. The timeline view permits an explicit identification of the evolution of different research domains. To investigate the properties of each cluster, a series of metrics, including temporal metrics (e.g., citation burst), structural metrics (e.g., betweenness centrality, modularity, silhouette score), and a combined concept of both metrics (also known as Sigma metrics) was adopted in CiteSpace. Citation burst is a concept corresponding to the circumstance in which a surge of citations of a particular publication occurs during a specific period [16]. If a cluster incorporates large amounts of nodes with high citation bursts, such a cluster may represent an emerging trend in current or future research. Betweenness centrality depends on the frequency a node lies on the shortest pathways between pairs of other nodes [17]. If a node was found to possess high betweenness centrality, it was considered a so-called turning point and marked with purple, with the color becoming brighter proportionally with increasing betweenness centrality [15]. For instance, papers regarded as turning points typically refer to those experiencing rapid growth in citations within a short period or serving as a milestone in the evolution of a specific research domain. The modularity score (the Q score), ranging from 0 to 1, is applied to infer whether a cluster can be explicitly distinguished [18]. A network with a Q score of 0.3 or higher typically possesses significant structure. The silhouette score (the S score), whose theoretical range was -1–1, was introduced to assess the quality of clustering analysis and data configuration [19]. An S score greater than 0.3, 0.5, and 0.7 is recognized as the major criteria for identifying the homogeneity, reasonability, and credibility of a network, respectively. Sigma is created based on merging betweenness centrality with citation burst ((betweenness centrality + 1)^citation burst^); therefore, such metric reflects both structural and temporal properties [20]. In addition, parameters used in CiteSpace were set as follows: (1) the period was from 2000 to 2022; (2) years per slice was 1; (3) pruning options including pathfinder, minimal spanning tree, pruning sliced networks, and pruning the merged network were enabled; (4) top number was 50; (5) the rest parameters retained default values.

## Results

### Analysis of co-cited references: a cluster of research and most cited papers

#### Cluster of research

We first constructed several cluster-based co-citation networks of retrieved references for 2000–2022, 2017–2022, and 2022, respectively. All three networks were validated to be well-structured and sufficiently credible (Q = 0.8329, S = 0.938 for the 2000–2022 network; Q = 0.7397, S = 0.9543 for the 2017–2022 network; and Q = 0.6672, S = 0.879 for the 2022 network, respectively). Each reference was represented by a single node, whose size was proportional to the times the reference has been co-cited. Detailed descriptions of the largest clusters of co-cited references were presented in Additional file 2: Table S1, and visualized networks of each identified cluster were illustrated in Additional file 1: Fig. S2. Moreover, we provided relevant information on the link walkthrough between clusters based on burst dynamics for the co-cited reference network (2000–2022) (Additional file 1: Fig. S3).

**Fig. 1:**
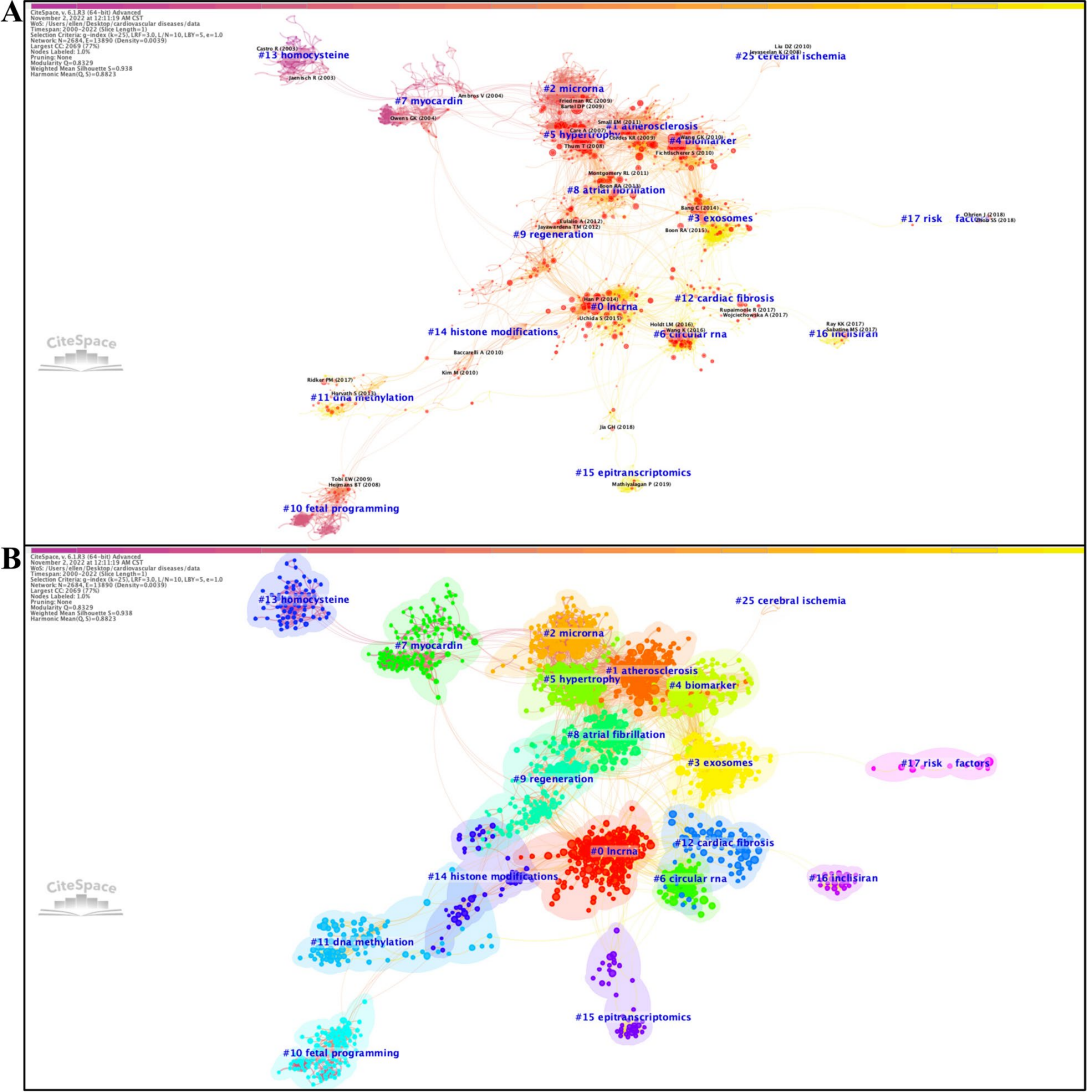
Co-citation network of references (2000–2022) with corresponding clusters obtained with CiteSpace. **A** Co-citation reference network with cluster visualization and citation bursts of hotspots. **B** Visualization map of the corresponding clusters and citation bursts of hotspots. Note: Each node represents a co-cited reference, with the size of the node being proportional to the number of times the reference has been co-cited. The tree rings surrounding the nodes refer to citation bursts of co-cited references

In the 2000–2022 co-citation network, we identified a total of 19 different clusters, each of which was assigned a cluster number that depends on their sizes (ranging from the largest size (#0) to the smallest size (#25)), along with other qualitative measures, including cluster label, cluster size (N), silhouette score (S), and mean year (Y) of co-cited references. To summarize how research topics in this field developed during the past 2 decades, we integrated these clusters into four major research trends: (a) epigenetic mechanisms of CVD, incorporating 9 clusters on “lncrna”, #0 (N = 223; S = 0.919; Y = 2015)”, on “microrna”, #2 (N = 198;S = 0.894; Y = 2005), on “circular rna”, #6 (N = 133; S = 0.983; Y = 201), on “myocardin”, #7 (N = 129; S = 0.981; Y = 2002), on fetal programming”, #10 (N = 105; S = 0.991; Y = 2005), on “dna methylation”, #11 (N = 80; S = 0.974; Y = 2015), on “homocysteine”, #13 (N = 69; S = 0.995; Y = 1999), on “histone modifications”, #14 (N = 57; S = 0.971; Y = 2008), and on “epitranscriptomics”, #15 (N = 38; S = 0.986; Y = 2018); (b) epigenetics-based therapies for CVD, incorporating 3 clusters on “exosomes”, #3 (N = 167; S = 0.947; Y = 2016), on “regeneration”, #9 (N = 120; S = 0.939; Y = 2011), and on “inclisiran”, #16 (N = 20; S = 0.998; Y = 2018); (c) epigenetic profiles of specific CVDs, incorporating 5 clusters on “atherosclerosis”, #1 (N = 209; S = 0.908; Y = 2011), on “hypertrophy”, #5 (N = 153; S = 0.907; Y = 2008), on “atrial fibrillation”, #8 (N = 122; S = 0.887; Y = 2012), on “cardiac fibrosis”, #12 (N = 72; S = 0.952; Y = 2018), on “cerebral ischemia”, #25 (N = 5; S = 1; Y = 2009); (d) epigenetic biomarkers for CVD diagnosis/prediction, incorporating only 2 clusters on “biomarkers”, #4 (N = 159; S = 0.923; Y = 2012) and on “risk factors”, #17 (N = 10; S = 0.995; Y = 2019) (Fig. 1).

In addition, we analyzed the co-cited references published from 2017 to 2022 (Additional file 1: Figs. S4 and S5) with yearly time slices and those published in 2022 with monthly time slices (Additional file 1: Fig. S6), which permitted an overview of the major research trends that emerged more recently. Not surprisingly, we found considerable overlap in identified clusters between this network and the 1989–2022 network, and determined 4 clusters that appeared for the first time during this period: (a) cluster #6 on “mir-29a” (N = 59; S = 0.96; Y = 2013); (b) cluster #7 on “heart failure” (N = 56; S = 0.972; Y = 2014); (c) cluster #9 on “RNA methylation” (N = 24; S = 0.989; Y = 2018); (d) cluster #12 on “transdifferentiation” (N = 14; S = 0.992; Y = 2013). As for the 2022 network, several novel clusters on “ferroptosis”, #0 (N = 84; S = 0.811; Y = 2019), on “clonal hematopoiesis”, #3 (N = 63; S = 0.897; Y = 2018), on “ischemic heart disease”, #4 (N = 48; S = 0.86; Y = 2018), on “cardiomyocyte proliferation”, #5 (N = 43; S = 0.896; Y = 2019), on “single-cell technology”, #9 (N = 13; S = 0.97; Y = 2019), on “brca1”, #10 (N = 11; S = 0.995; Y = 2019), on “somatic cell reprogramming”, #11 (N = 10; S = 1; Y = 2018), on “angiotensinogen”, #12 (N = 10; S = 0.989; Y = 2019), on “twin-twin transfusion syndrome”, #13 (N = 8; S = 0.985; Y = 2018), and on “dex”, #14 (N = 5; S = 0.993; Y = 2018) was noted, representing the most attractive topics at present.

#### Most cited papers

We extracted the top 10 papers with the highest citation frequencies published during the period 2000–2022 (Table 1). Of all these papers, those ranking within the top 3 positions were David P. Bartel’s review on the current status of the knowledge of miRNA target recognition in mammals and the mechanisms whereby miRNA modulates the expression and activity of protein-coding genes (337 citations) [21], followed by Carè et al. work establishing the essential role of miR-133 in suppressing cardiac hypertrophy in vitro and in vivo (276 citations) [22], and Thum et al. research article that an upregulated cardiac fibroblast-specific miR-21 expression was observed in pressure overload-induced failing myocardium, and may further exert detrimental effects on the geometry and function of heart through activating extracellular signal-regulated kinase–mitogen-activated protein kinase (ERK–MAPK) signaling pathway (270 citations) [23].

**Table 1:** The top 10 most cited references

Number of citations in the network	Number of citations in the literature	Cited reference	Year	Source	Vol	Page	Title	Doi	Type of paper	Related cluster in Fig. 1
337	15,077	Bartel DP	2009	CELL	136	215	MicroRNAs: Target Recognition and Regulatory Functions	10.1016/j.cell.2009.01.002	Review	2
276	1,413	Care A	2007	NAT MED	13	613	MicroRNA-133 controls cardiac hypertrophy	10.1038/nm1582	The Basic Research	5
270	1,823	Thum T	2008	NATURE	456	980	MicroRNA-21 contributes to myocardial disease by stimulating MAP kinase signalling in fibroblasts	10.1038/nature07511	The Basic Research	5
269	1,282	Van ROOIJE	2007	SCIENCE	316	575	Control of stress-dependent cardiac growth and gene expression by a microRNA	10.1126/science.1139089	The Basic Research	5
268	962	Fichtlscherer S	2010	CIRC RES	107	677	Circulating MicroRNAs in Patients With Coronary Artery Disease	10.1161/CIRCRESAHA.109.215566	The Basic Research	4
260	920	Wang GK	2010	EUR HEART J	31	659	Circulating microRNA: a novel potential biomarker for early diagnosis of acute myocardial infarction in humans	10.1093/eurheartj/ehq013	The Basic Research	4
258	930	Small EM	2011	NATURE	469	336	Pervasive roles of microRNAs in cardiovascular biology	10.1038/nature09783	Review	1
243	1,427	Van ROOIJE	2008	P NATL ACAD SCI USA	105	13,027	Dysregulation of microRNAs after myocardial infarction reveals a role of miR-29 in cardiac fibrosis	10.1073/pnas.0805038105	The Basic Research	5
241	1,220	Van ROOIJE	2006	P NATL ACAD SCI USA	103	18,255	A signature pattern of stress-responsive microRNAs that can evoke cardiac hypertrophy and heart failure	10.1073/pnas.0608791103	The Basic Research	5
225	763	Creemers EE	2012	CIRC RES	110	483	Circulating MicroRNAs Novel Biomarkers and Extracellular Communicators in Cardiovascular Disease?	10.1161/CIRCRESAHA.111.247452	RCT	4

Moreover, we analyzed the impact of papers published during the period 2000–2022 and 2017–2021, respectively, using calculating the citation bursts (Additional file 3: Supplementary Tables S2S–V). The blue line is the timeline sliced year by year, and the red line is representative of how long a citation burst persists. The top 3 references with the latest and strongest beginning of citation bursts included “A circular RNA protects the heart from pathological hypertrophy and heart failure by targeting miR-223” published by Wang et al. in 2016 [24], “A long noncoding RNA protects the heart from pathological hypertrophy” published by Han et al. in 2014 [25], and “Circulating microRNAs: novel biomarkers and extracellular communicators in cardiovascular disease?” published by Creemers et al. in 2012 [26]. As for the last 5 years, the top 3 references were an updated report on the epidemiological statistics of CVDs in the USA launched by the American Heart Association (AHA) [27], Love et al. article that introduced a sophisticated R package, namely, DESeq2, for handling with count data produced in high-throughput sequencing assay, which has currently become one of the most widely used techniques in determining CVD-related epigenetic loci [28], and another research article demonstrating the utmost importance of a highly conserved lncRNA metastasis-associated lung adenocarcinoma transcript 1 (MALAT1) for enabling the normal function of vascular endothelial cells and stimulating angiogenesis [29].

### Analysis of co-occurrence of keywords

**Fig. 2:**
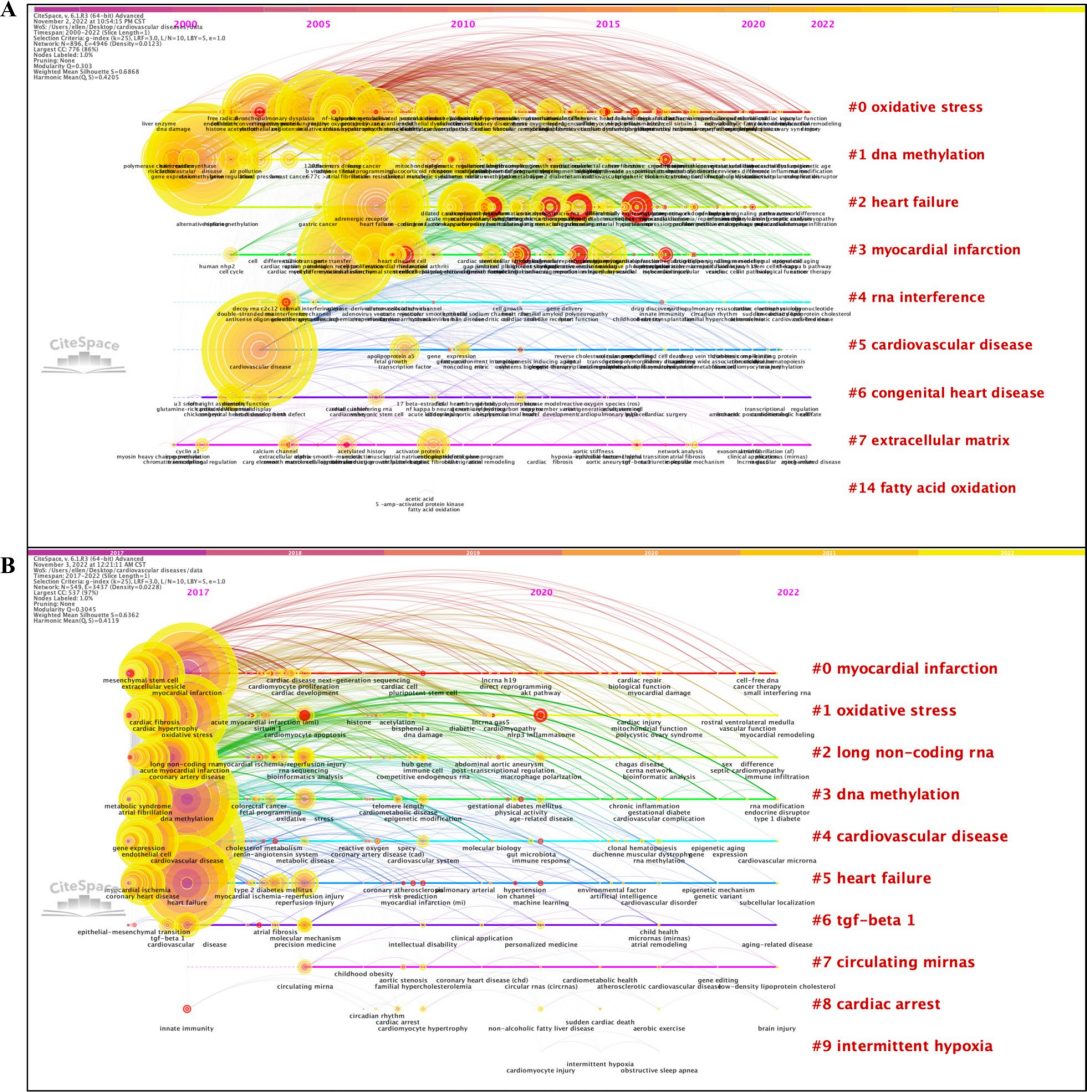
Timeline visualization of networks of co-occurring keywords for the period 2000–2022 (**A**) and 2017–2022 (**B**). Note: Each node is representative of a co-occurring keyword, with the color of the node depending on the average publication year of all articles containing this keyword. A brighter node refers to a keyword that emerged more recently. The tree rings surrounding the nodes represent citation bursts of co-occurring keywords. The networks are weighted on total link strength across different nodes and scored on the average publication years. The identified cluster labels are marked in red, and placed on the right side of the networks

The predominant goal of constructing co-occurrence networks of keywords is to gain a comprehensive overview of the current research status and to forecast the evolution of research hotspots over time. A single node in this network is indicative of a highly co-occurring keyword, with the node size depending on how frequently it occurs. The 2000–2022 and 2017–2022 networks both exhibited significant structure and adequate reasonability (Q = 0.303, S = 0.6868 for the 1989–2022 network; and Q = 0.3045, S = 0.6362 for the 2017–2022 network, respectively) (Fig. 2).

In the 2000–2022 network, we identified 9 distinct clusters: cluster #0, “oxidative stress”, cluster #1, “DNA methylation”, cluster #2, “heart failure”, cluster #3, “myocardial infarction”, cluster #4, “RNA interference”, cluster #5, “cardiovascular disease”, cluster #6, “congenital heart disease”, cluster #7, “extracellular matrix”, and cluster #14, “fatty acid oxidation”. When focusing on the last 5 years, we found several newly emerging clusters: cluster #2, “long non-coding RNA”, cluster #6, “the-beta 1”, cluster #7, “circulating miRNAs”, cluster #8, “cardiac arrest”, and cluster #9, “intermittent hypoxia”.

Keywords with the latest and highest citation bursts are highly predictive of hotspot topics, research frontiers, and future research trends. The top 3 keywords that appeared most recently and had the strongest beginning of citation bursts included “nlrp3 inflammasome”, “myocardial injury”, and “reperfusion injury” for the 2000–2022 network, while those for the 2017–2022 network were “machine learning”, “nlrp3 inflammasome”, and “risk prediction” (Additional file 3: Tables S2W–Z).

In addition, we employed VOSviewer to establish a network visualization of co-occurring keywords, in which the lines connecting pairs of keywords become thicker with increasing numbers of co-occurrences. An overlay visualization of co-occurring keywords—wherein one keyword was assigned a specific color that varies from blue to yellow depending on the mean years of publications of articles incorporating this keyword (keywords occurring in earlier years were colored in blue, and those appearing later were colored in yellow)—was also provided. Interestingly, despite five distinct clusters being screened out and marked with different colors, all these clusters were exceptionally similar in the evolving process of research trends, and an even distribution of newly emerging topics across the clusters was noted (Additional file 1: Fig. S7).

### Publication outputs and major journals

We originally retrieved 30,180 articles regarding epigenetics in CVDs from the WoSSC database, ruled out 2418 non-relevant ones, and included the rest for further analyses (Additional file 1: Fig. S1). The yearly amounts of publications displayed an exponential rise since the early 2000s, and the last 5 years have witnessed a surge of publications, implying a rapidly expanding interest in this realm. During the same period, the average number of citations per year also tended to increase gradually, reached its peak in 2005, and then slightly declined but still maintained at a relatively high level; however, the entire course seemed to be more fluctuating (Additional file 1: Fig. S8).

From 2000 to 2022, the leading journal that published the most references was Scientific Reports, followed by the International Journal of Molecular Sciences and Circulation Research. For the last 5 years, the International Journal of Molecular Sciences, Scientific Reports, and Frontiers in Cardiovascular Medicine constituted the top 3 journals with the highest publications. Most journals displayed an approximately linear increase in the number of publications, while the International Journal of Molecular Sciences and Frontiers in Cardiovascular Medicine were the only two that experienced an inflection point during this period. The rising speed of the number of publications on the right side of the inflection point was higher compared to that on the other side of the inflection point, causing more rapid growth of publications in these two journals (Additional file 1: Fig. S9). An overlay visualization of the most cited journals over the past 5 years and a co-citation network of journals for the past 2 decades were also presented (Additional file 1: Fig. S10).

The top 3 journals with the latest and strongest beginning of citation bursts for the past 2 decades were Frontiers in Immunology, Frontiers in Genetics, and Bioscience Reports (Additional file 3: Tables S2G, H), while those for the last 5 years were Artificial Cells, Nanomedicine, and Biotechnology, Clinical Medicine Insights: Cardiology, and Brain and Behavior (Additional file 3: Tables S2I, J).

### Analysis of cooperation network across countries and institutions

**Fig. 3:**
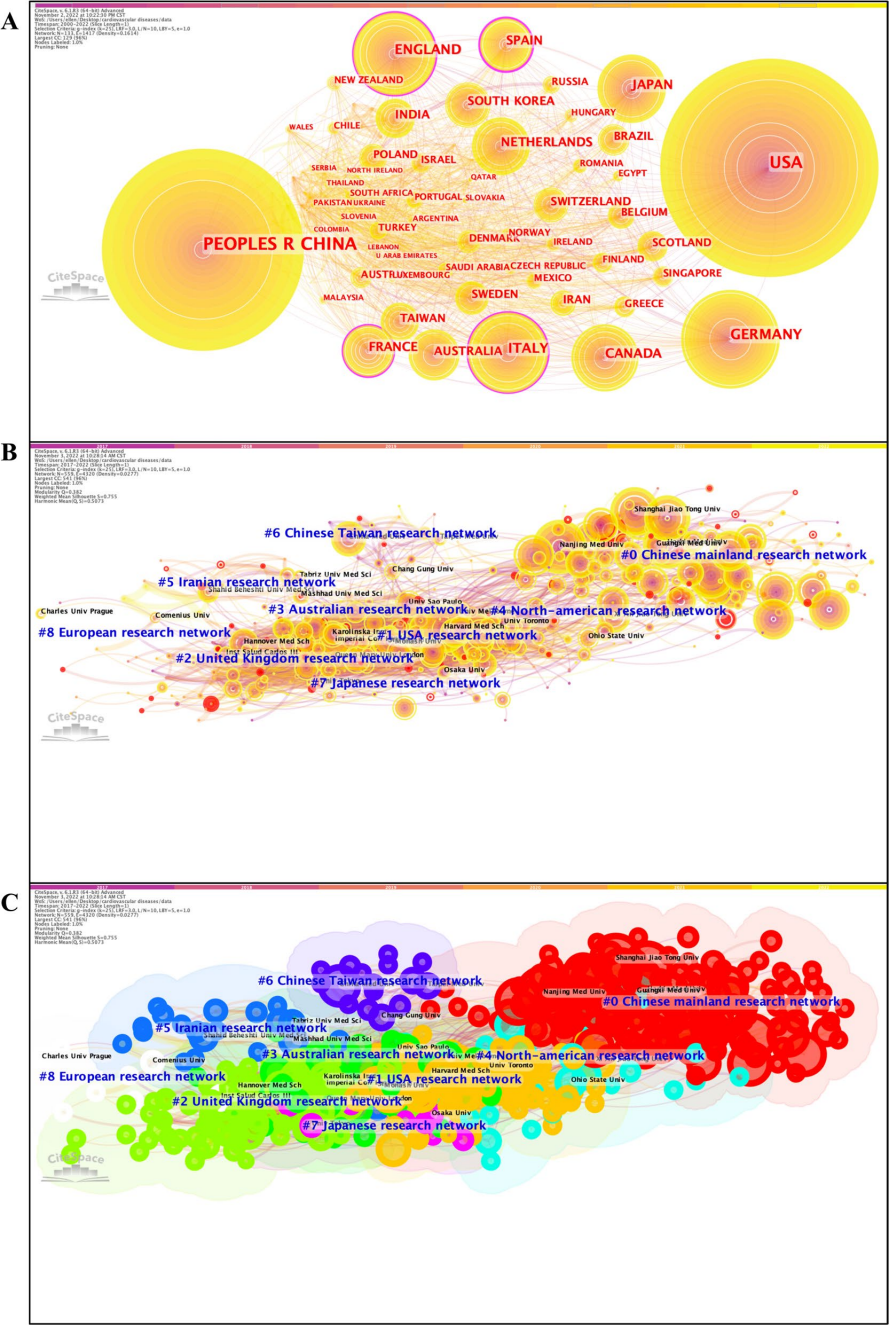
Co-citation network of co-authors’ countries (2000–2022) (**A**) and co-citation network of co-authors’ institutions (2017–2022) (**B**) with corresponding clusters (**C**)

We constructed the co-citation networks of countries and institutions (Fig. 3), and listed the top countries and institutions ranked by number of citations and betweenness centrality (Additional file 6: Table S5). China made the most prominent contribution to citation counts (n = 9910), followed by the United States (n = 8271), and Germany (n = 1984). Spain ranked first in betweenness centrality (0.23), followed by England (0.12), and France (0.11). When focusing on the last 5 years, the top 3 positions in the rankings of citation numbers remained unchanged, whereas France began to surpass England and Spain and became the area with the highest degree of betweenness centrality.

The top 3 institutions ranked by citation numbers were Nanjing Medical University (n = 472), Harbin Medical University (n = 439), and Shanghai Jiao Tong University (n = 428). In contrast, those ranked by betweenness centrality were Harvard University (0.11), Baylor College of Medicine (0.06), and Columbia University (0.04). The top 3 most cited institutions identified within the last 5 years were quite similar, except Harvard Medical School. In terms of betweenness centrality, the University of Medical Center Utrecht (0.08) achieved the best performance during this period, followed by the University of Texas Health Science Center (0.05) and the University of Naples Federico II (0.05).

Moreover, the top 3 countries that possessed the latest and highest citation bursts over the period 2000–2022 included Russia, Pakistan, and South Africa (Additional file 3: Tables S2A, B). As for the institutions, Central South University and Chinese Academy of Medical Sciences & Peking Union Medical College, and Shandong First Medical University retained leading positions for either the 2000–2022 period or the 2017–2022 period (Additional file 3: Tables S2C–F).

### Analysis of co-authorship network

**Fig. 4:**
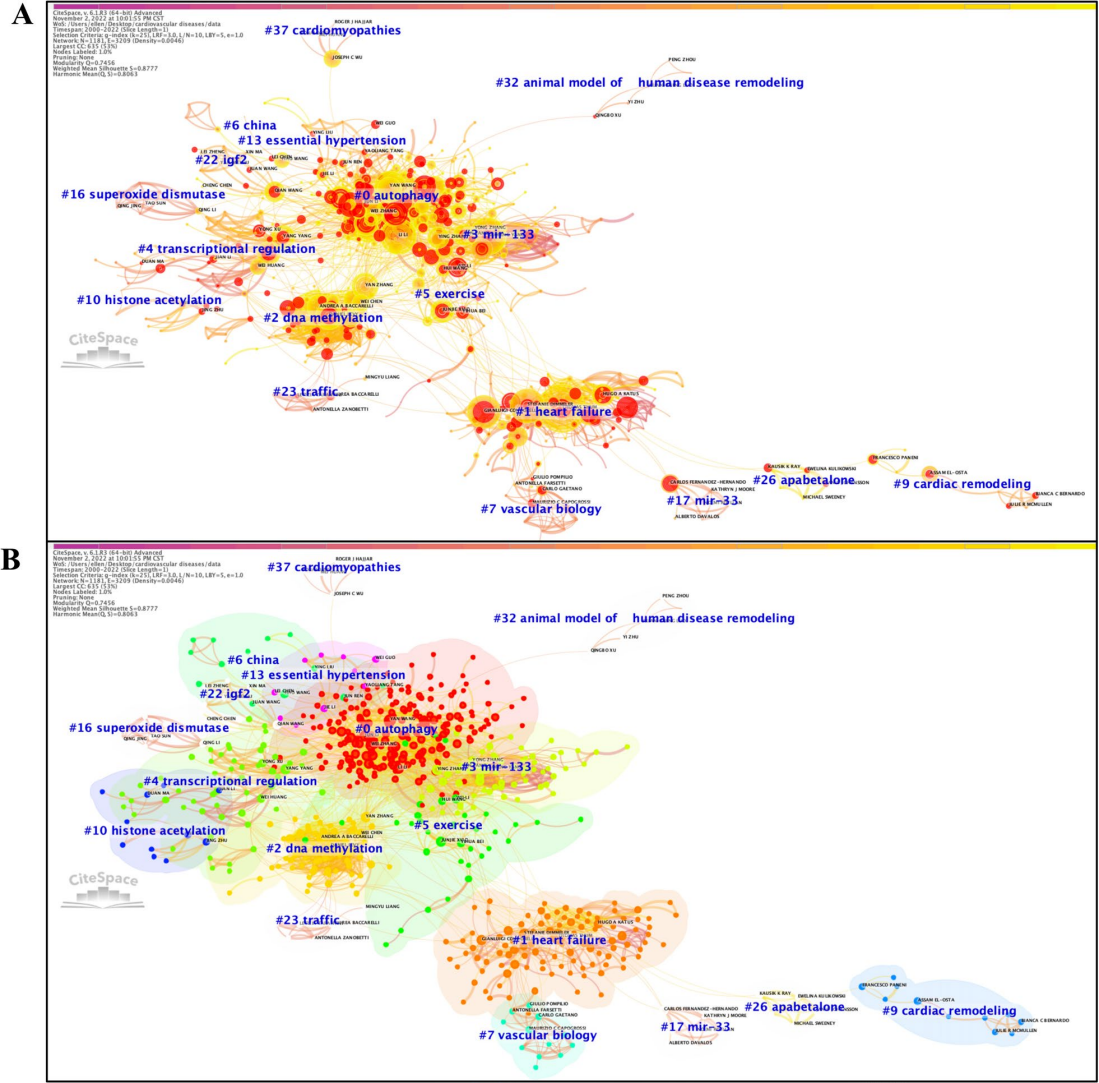
Co-authorship network (**A**) with corresponding clusters (**B**) for the period 2000–2022

To analyze and visualize the collaboration between different researchers based on the amount of co-authored publications, a well-structured and highly credible co-authorship network (Q = 0.7456, S = 0.8777) was thus established via CreateSpace (Fig. 4). In this network, we found the three most important clusters included: cluster #0, “autophagy”, cluster #1, “heart failure”, cluster #2, and “DNA methylation” (Additional file 4: Table S3). According to the findings of the burst analysis, the top 3 co-authors considered the most recent and influential contributors from 2000 to 2022 were Liu Y, Wang J, and Zhang J (Additional file 3: Tables S2K, L), and those for the 2017–2022 period were Katus HA, Wang Y, and Wang X (Additional file 3: Tables S2M, N). In addition, a similar co-authorship network was obtained with VOSviewer (Additional file 1: Fig. S11).

We further investigated the co-citation relations among authors from 2017 to 2022 (Additional file 1: Fig. S12). The top 3 co-cited authors who possessed the latest and strongest citation bursts were Bonauer A, Small EM, and Fichtlscherer S for the period 2000–2022 (Additional file 3: Tables S2O, P), and were Bolli R, Zhu HY, and Zhang XQ for the period 2017–2022 (Additional file 3: Tables S2Q, R, Additional files 5 and 6).

### Bibliographic coupling analysis of countries, institutions, journals, references, and authors

**Fig. 5:**
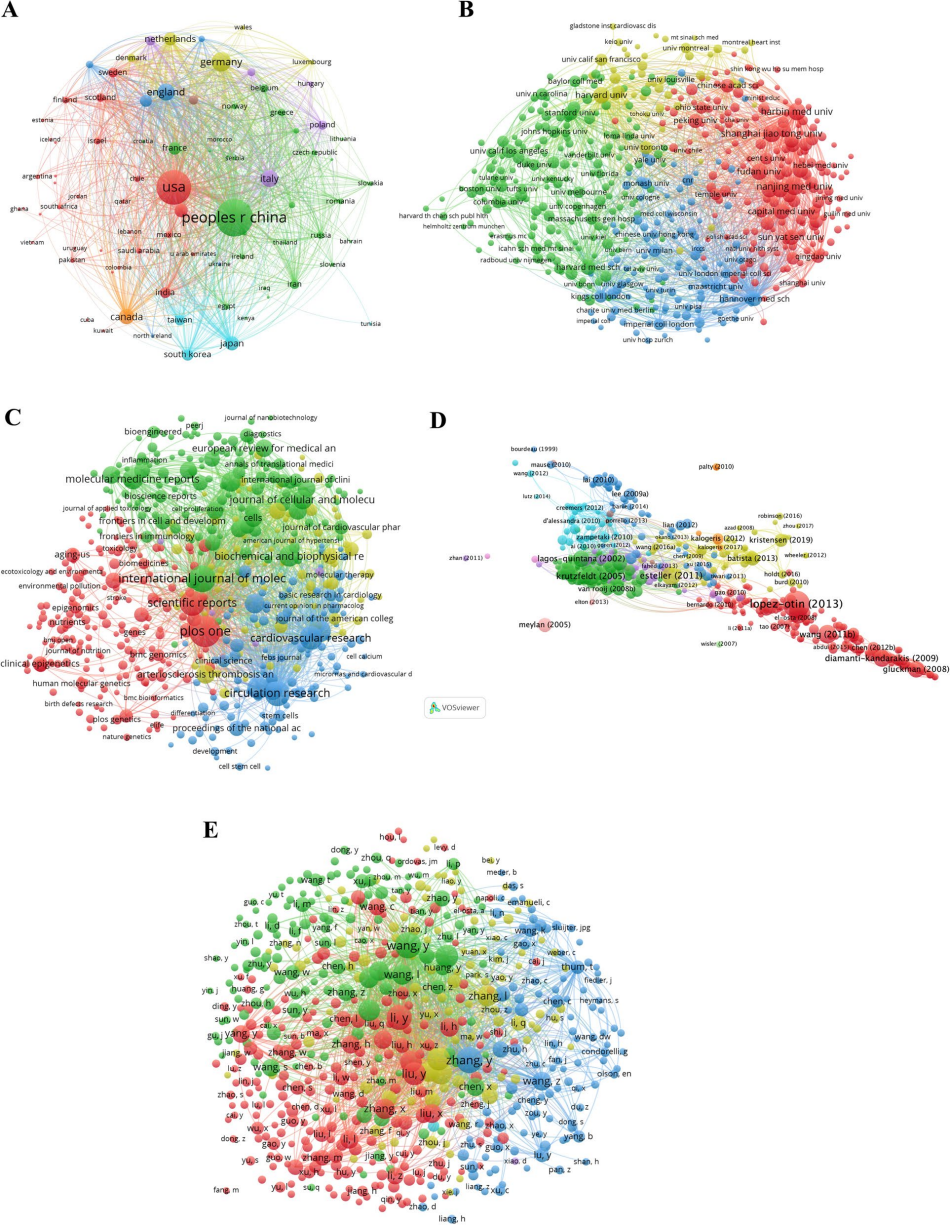
Bibliographic coupling networks of countries (**A**), institutions (**B**), journals (**C**), references (**D**), and authors (**E**) (weighted on the total link strength). Note: The minimum number of documents in a country should exceed 583; the Minimum number of citations of a document should exceed 240,490; the Minimum number of documents of a journal should exceed 10,519; Minimum number of documents of an author should exceed 30,579; Minimum number of documents of an institution should exceed 25,507

Next, we employed VOSviewer to examine the bibliographic coupling of the publications in terms of different countries/institutions/authors/journals/references (Fig. 5), and calculated the total link strength in the bibliographic coupling networks to illuminate the relatedness of research domains (Additional file 7: Table S6). Among all countries, the United States had the strongest total link, followed by China and Germany. As for institutions, Hannover Medical School occupied the top position, followed by Harbin Medical University and Harvard University. Then, the top 3 journals that possessed the largest total link strength included International Journal of Molecular Sciences, Plos One, and Circulation Research, and the top 3 references were: “Non-coding RNAs in Development and Disease: Background, Mechanisms, and Therapeutic Approaches” published by Beermann et al. in 2016 [30], “MicroRNA regulatory networks in cardiovascular development” published by Liu et al. in 2010 [31], and “MicroRNAs add a new dimension to cardiovascular disease” published by Small et al. in 2010 [32]. Finally, Wang Y, Zhang Y, and Li Y constituted the three authors that performed best in total link strength.

## Discussion

### Summary of the main findings

Herein, we depicted a comprehensive landscape of global research on epigenetics in CVD produced during the past 2 decades, with specific attention on analyzing the current research status, summarizing major research trends, and predicting future hotspots and frontiers. We found the yearly number of publications started to continuously increase since the early 2000s and is promising to maintain a rising trend in the future. China, the United States, and Germany comprised the top 3 most cited countries, while Spain was the most cooperative country, followed by England and France. Nanjing Medical University, Harbin Medical University, and Shanghai Jiao Tong University were the 3 institutions with the highest citation frequencies, whereas Harvard University, Baylor College of Medicine, and Columbia University had the most crucial roles in promoting international cooperation. The most recent and strongest citation bursts were found in Frontiers in Immunology, followed by Frontiers in Genetics and Bioscience Reports. As for the most influential co-authors, the top 3 first authors with the latest and strongest citation bursts were Bonauer A, Small EM, and Fichtlscherer S. We also explored the similarities in the research topics by performing bibliographic coupling analysis of the publications belonging to different countries, institutions, journals, or authors.

### Identification of research trends

As we mentioned above, 19 identified clusters of the co-citation network of references (2000–2022) were categorized into several research trends, and a couple of clusters that were not highlighted in the 2000–2022 network were presented for the first time in either the 2017–2022 network or the 2022 network, which may represent the newest research focuses of these trends.

The first and largest trend that focuses on the epigenetic mechanisms involved in CVD pathogenesis started around the first decade of the 2000s when a biological phenomenon in which a series of adaptive epigenetic variations induced by undesirable in-utero environment (also known as fetal programming) was discovered to be closely related to the vulnerability to CVD in adulthood (cluster #10, “fetal programming”) [33]. During the same period, the link between hyperhomocysteinemia (HHcy) and elevated CVD risk was also noted and was further demonstrated to be partially attributed to homocysteine-induced epigenetic modifications (cluster #13, “homocysteine”) [34]. Subsequent identification of myocardin as a cardiac- and smooth muscle-enriched transcriptional coactivator of serum response factor (SRF), a class of transcription factors involved in the regulation of multiple biological processes, and the establishment of its function in modulating cardiac gene expression determined the important status of epigenetic factors in heart development and post-natal cardiac remodeling (cluster #7, “myocardin”) [35]. Based on these situations in which epigenetic regulation plays an essential role in promoting CVD, different epigenetic regulatory patterns, especially DNA methylation (cluster #11, “DNA methylation”) [36] and histone modifications (cluster #14, “histone modifications”) [37], were gradually uncovered and became hotspot topics in CVD research at that time. Later, arising interest was aggregated on the issue of posttranscriptional regulation of CVD-related genes by ncRNAs-predominantly incorporating miRNAs (cluster #2, “microRNA”) [38], lncRNAs (cluster #0, “lncRNA”) [39], and circRNAs (cluster #6, “circular RNA”) [40]—in a short period, which also prompted the emergence of a novel cluster that concerns CVD-related epi-transcriptomic profiles (cluster #15, “epi-transcriptomics”) [41], whereby an overview of epi-transcriptomic alternations under cardiac pathological conditions can be detected across multiple dimensions after the revolutionary leap in sequencing technologies.

The second research trend focusing on epigenetics-based therapies for CVD incorporated three major clusters. Cluster #9 on “regeneration” concerns the double-edged role of epigenetic mechanisms in post-injury cardiac regeneration emerged as the beginning of this trend [42]. Subsequently, the potential of exosomes—commonly defined as nanosized extracellular vesicles (EVs) that can serve as endogenous carriers of biologically active molecules, especially different types of ncRNAs, for intercellular communication—to be developed into promising therapeutic targets rendered these substances exceptionally attractive in CVD treatment, and gave rise to a surge of publications on this topic (cluster #3, “exosomes”) [43]. The latest cluster of this trend was formed based on the wide application of inclisiran, a first-in-class proprotein convertase subtilisin/kexin type 9 (PCSK9)-targeted small interfering RNA (siRNA) for lipid-lowering therapy, and the determination of its prominent effects on reducing long-term cardiovascular risk in clinical practice (cluster #16, “inclisiran”) [44].

The third research trend was centered around epigenetic alternations underpinning various CVDs. This trend originated from a cluster on cardiac hypertrophy (cluster #5, “hypertrophy”) [45], predominantly due to its high prevalence in the context of multiple cardiac pathological conditions and its close relation with epigenetic modifications, followed by two distinct clusters, namely, cluster #8 on “atrial fibrillation” [46] and cluster #1 on “atherosclerosis” [47] during virtually the same period. With the accumulation of knowledge about how epigenetic effectors participate in the initiating cardiac fibrotic events, this trend continued to evolve into a cluster of cardiac fibrosis, which is one of the most important hallmarks of end-stage CVDs and is commonly predictive of poor prognosis in CVD patients (cluster #12, “cardiac fibrosis”) [48]. More recently, we observed the formation of a tiny but unneglectable cluster concentrating on the epigenetic etiologies of cerebral ischemic diseases, especially stroke (cluster #25, “cerebral ischemia”) [49].

The rationale for the formation of the fourth trend was an urgent need to achieve the goal of diagnosing/predicting CVD quickly and effectively via an epigenetic approach. Unlike the other research trends, this trend occurred relatively late, starting with cluster #4 on “biomarker”, in which the application of epigenetic biomarkers in assisting the diagnosis of cardiac fibrosis was comprehensively assessed [50]. The advent of the latest cluster on “risk factors”, #17 around the 2020s indicated the mounting interest in the question of how to identify high-risk populations for CVD by analyzing epigenetic predictors and may therefore provide profound insights into the preventative strategies for CVD [51].

The novel clusters that emerged within the last 5 years predominantly concerned some novel mechanisms, predictive methods, and therapeutic strategies for CVD from an epigenetic perspective, including cluster #6 on “mir-29a” that assessed the potential of miR-29 as a reliable circulating biomarker for CVDs [52], especially cardiac hypertrophy and fibrosis, cluster #9 on “RNA methylation” in which mounting attention was attached to RNA methylation, another kind of posttranscriptional epigenetic modification catalyzed by RNA methylase that persistently happens in response to adverse cardiovascular stimulus [53], and cluster #12 on “transdifferentiation” that discussed the feasibility of the clinical application of cardiac fibroblast-to-cardiomyocyte conversion for evoking cardiac regenerative capacity [54], as well as another cluster investigating the epigenetic alterations linked with the pathological remodeling of the failing heart (cluster #7, “heart failure”) [55]. When focusing on the last year, a prominent feature of global studies during this period is the back-to-back appearance of a couple of clusters with respect to atherosclerotic cardiovascular disease (ASCVD), with specific attention on epigenetic changes prior to observable ischemic lesions (cluster #4, “ischemic heart disease”) [56], on the link between clonal hematopoiesis (CH) and ASCVD mediated by unexpected somatic mutations in genes encoding ASCVD-related epigenetic regulators (e.g., DNA methyltransferase 3 alpha (*DNMT3A*)) (cluster #3, “clonal hematopoiesis”) [57], on single-cell RNA sequencing (scRNA-seq) employed for decoding cell-specific epigenomes in the setting of ASCVD (cluster #9, “single-cell technology”) [58], and on some promising epigenetics-based therapies that may promote cardiomyocyte proliferation or direct reprogramming of non-cardiomyocytes into cardiomyocytes, thus supplying a supplement to the pools of functional cardiomyocytes (cluster #5, “cardiomyocyte proliferation”; cluster #11, “somatic cell reprogramming”) [59, 60]. The epigenetic modulators that have an impact on ferroptosis, also known as a new form of iron-dependent cell death with well-documented roles in CVD occurrence, became the most popular research topic, thereby rendering relevant publications the largest cluster in the 2022 reference co-citation network (cluster #0, “ferroptosis”) [61]. The successful use of liver angiotensinogen-targeted small interfering RNA (siRNA) in long-term anti-hypertensive treatment has placed RNA interference under the spotlight for dealing with CVD (cluster #12, “angiotensinogen”) [62]. Moreover, three smaller clusters were also significant, which detected whether epigenetically modified breast cancer-related *BRCA1* loci moonlights as a potential CVD biomarker (cluster #10, “brca1”) [63], investigated the long-term effects led by fetal programming on the cardiovascular health outcomes in adulthood of infants surviving from twin-twin transfusion syndrome (TTTS) (cluster #13, “twin-twin transfusion syndrome”) [64] and focused on the unique epigenetic mechanisms underpinning the cardioprotective property of dexmedetomidine (Dex) (cluster #14, “dex”) [65], respectively.

To consolidate the findings drawn from the analyses of co-cited references, citation bursts of keywords can be used to reflect the latest research trends and aid in the identification of research frontiers. A keyword with the most recent and strongest beginning of citation bursts is commonly a hallmark of a hotspot research topic. NLR family pyrin domain containing 3 (NLRP3) inflammasome activation is the leading cause of aberrant immune response and systemic inflammation predisposing to CVD, and manipulating the expression and activity of NLRP3 mRNA via epigenetic approaches therefore acts as one of the most attractive research topics (“nlrp3 inflammasome”). Our results also revealed accumulating interest in delineating the complex roles of epigenetic modifications in myocardial ischemia/reperfusion (I/R) injury (“myocardial injury”, “reperfusion injury”) and new studying approaches established upon either machine learning algorithms or numeric models to early predict the risk of developing CVD (“machine learning”, “risk prediction”) (Additional file 3: Tables S2W–Z).

Notwithstanding the remarkable advancements in the realm of epigenetics of CVD, further research is warranted to bridge two knowledge gaps: (a) plenty of epigenetic effectors with known detrimental effects on the cardiovascular system have been identified to date, whereas available data on what epigenetic alternations happens to different cellular components of cardiac or vascular tissues when CVD occurs is relatively sparse. Luckily, the advent of single-cell and spatial sequencing techniques allows researchers to map the epigenetic alternations in specific genes in terms of an individual cell or an anatomical region of interest. In this case, applying these methods to gain a more in-depth understanding of the epigenetic origin of CVDs and conducting more gain- and loss-of-function experiments using some state-of-art epigenetic editing technologies (e.g., Cre/loxP, CRISPR/Cas9) to realize cell-specific epigenetic modulation of hub genes implicated in CVD development is urgently needed; (b) as demonstrated by our findings, basic research and review articles account for the vast majority of the most cited references (Table 1). In contrast, influential clinical trials and practice guidelines are quite lacking, which seemingly reflects that the wide application of epigenetically targeted therapies from bench to bedside for treating CVD has not yet been fully achieved at present. Indeed, genetic or drug-induced rodent models of CVD are indispensable tools for examining the therapeutic efficacy and safety of pharmacologically intervening epigenetic effectors, these animal models, however, to a large extent cannot recapitulate the pathophysiologic complexity of CVD in human patients, which could make sense as to why we should shift attention to human-based models or perform more clinical trials to overcome the translational barriers.

### Practical significance of bibliometric studies

There are several aspects that researchers can benefit from visualized bibliometric studies [8]. First, reference co-citation analysis permits a comprehensive science mapping of global research on epigenetics in CVD generated during the past decades, specifically the clear identification of the birth, development, and vanishment of a research topic throughout a long study period (Fig. 1), and analyses of co-occurring keywords and citation burst of keywords would help to optimize the keyword-based database searching strategies when selecting eligible papers for systematic reviews and meta-analyses [66] (Fig. 2), and to highlight newly emerging frontiers and hotspots that deserve greater attention at present, respectively (Additional file 3: Tables S2W–Z). Then, the collaboration network is generated by assessing co-cited relations between countries and institutions that have made contributions to this field (Fig. 3). Combined with the visualized clusters extracted from the co-citation reference networks, researchers can easily capture useful information on research teams with similar or overlapping areas of interest and thereby evaluate possible candidates for more collaborations. For instance, although CVD poses an overwhelming threat to all human beings regardless of countries/regions and races/ethnicities, a surprising finding we have drawn from the analyses of global impact and cooperation is that the international and inter-institutional collaboration among the greatest contributors to research on epigenetics in CVD (including the United States, China, and some European countries) is far from adequate at present and needs to be reinforced in the face of the impending challenge of CVD. Furthermore, the determination of leading journals in this field can provide key information on which journal researchers should consider as a priority for submitting their research papers and presenting their findings therein (Additional file 1: Fig. S10).

### Strengths and limitations

To the best of our knowledge, we made an up-to-date report of global publications on epigenetics in CVDs using bibliometric methods to answer the question of what changes have taken place in this field during the past 2 decades and which direction current research focuses may evolve towards in the future. The most prominent advantage of our study is that we used several sophisticated analytical tools (Bibliometrix, VOSviewer, and CiteSpace) to interpret major bibliometric characteristics (co-citation, co-occurrence, co-authorship, and bibliographic coupling) and generate informative visualized networks in which relatedness between item pairs can be simplified as spatial distribution of nodes; therefore, such approach may assist the identification of evolving research trends, frontiers, and hotspots over time, present a comprehensive science map of the contemporary research status in this field to medical researchers and clinicians worldwide, and pave the way for advancing future medical research and formulating appropriate therapeutic strategies. Moreover, compared to those lagging or “old-fashioned” narrative reviews that are inevitably disturbed by man-made selection bias, this research pattern enables us to inspect the evolving process of a research theme that appears during a certain period in a more real-time and unprejudiced manner.

On the other hand, some limitations still require to be admitted and resolved later: (a) WoSSC was the only data source in the present study, which may cause a potential incompleteness of data acquisition. Other online databases (e.g., Scopus, Pubmed, Embase) are also candidate choices for bibliometric analysis, while some key publication information (e.g., full text, citation count) was not always accessible, making it difficult to integrate the data downloaded from these databases with those acquired from WoSSC database. (b) We only retained original articles and reviews (including early access) for further analyses; therefore, we cannot recapitulate the entire characteristics of all publications in this field. In other words, caution should be taken during the analysis and interpretation of the data; (c) When an author intends to express his/her own opinions based on already established foundations, decisions on citing references should be made prudently to reflect the authentic link between studies conducted at different stages and guarantee the reliability of citations; however, it may sometimes be distorted by far-fetched or inappropriate citations of non-relevant documents, leading to another type of bias in which careless readers may be misled by statements without solid evidence [67]. (d) Given only the first author was taken into account when performing the co-citation analysis of authors, how to achieve the goal of assessing the influence of each co-author remains a tough task.

## Conclusion

In summary, we performed a bibliometric study of global publications on epigenetics in CVD throughout the past 2 decades (2000–2022). The annual number of publications has grown rapidly during this period. The most influential and cooperative countries, institutions, and authors were determined, and the latest hotspot topics were extracted by analyzing citation bursts of keywords. More importantly, we identified four major research trends, including epigenetic mechanisms of CVD, epigenetics-based therapies for CVD, epigenetic profiles of specific CVDs, and epigenetic biomarkers for CVD diagnosis/prediction, and witnessed the evolution of these trends over time. China and the United States are predominant sources of publication impacts, whereas more cooperation between institutions from China and those from the United States and Europe is expected in the future. These results may provide important information for medical researchers and clinical practitioners to better understand the evolving trends, frontiers, and hot topics in CVD research from an epigenetic perspective.

### Supplementary Information

**Additional file 1**. **Figure S1**: Flow chart of the bibliometric study. **Figure S2**: Detailed information on the most important clusters of the co-citation network of references ranked by citation bursts for the period 2000–2022. For each cluster, we listed the top five keywords and labeled the cluster with the most-cited keyword (generated by comparing the likelihood ratio of keywords). These keywords are highly predictive of the overall topic of a cluster. The citation burst of each cluster is represented by the tree rings surrounding the nodes. **Figure S3**: Link walkthrough between clusters based on citation bursts for the co-citation network of references (2000–2022). **Figure S4**: Co-citation network of references (**A**), corresponding clusters (**B**), and timeline visualization of the network (**C**) for the period 2017–2022. **Figure S5**: Detailed information on all 17 extracted clusters of the co-citation network of references ranked by citation bursts for the period 2017–2022. **Figure S6**: Co-citation network of references (**A**), corresponding clusters (**B**), and timeline visualization of the network (**C**) for the year 2022. **Figure S7**: Overlay visualization of the co-occurring network of keywords (2000–2022) (**A**) with scores based on the average publication year (**B**). Note: The minimum number of keyword co-occurrences should exceed 51,000. Each node represents a co-occurring keyword, with the size of the node being proportional to the frequency of keyword co-occurrence and the color of the node varying from blue to yellow depending on the average publication year of articles incorporating this keyword (keywords occurring in earlier years were colored in blue, and those appearing later were colored in yellow). The co-occurrence network is weighted on total link strength across different nodes, which is scored based on the average publication year. **Figure S8**: Annual scientific production (**A**) and average citation per year for references (**B**) (2000–2022). **Figure S9**: The top 10 growth sources of publications (2000–2022 and 2017–2022). **Figure S10**: Overlay visualization of the most cited journals during the last five years (**A**), and the most co-cited journals that published the most articles during the last 20 years (**B**). **Figure S11**: The co-authorship network was obtained with VOSviewer. A total of 24 clusters that comprise 875 different authors were identified. Minimum number of articles of an author should exceed 13. **Figure S12**: Co-citation network of authors (**A**), corresponding clusters (**B**), and timeline visualization of the network (**C**) for the period 2017–2022.

**Additional file 2**. **Table S1**: Detailed information on the major clusters extracted from the co-citation networks of references (2000–2022, 2017–2022, and 2022).

**Additional file 3**. **Table S2**: Citation burst analysis of countries, institutions, authors, references, and keywords (2000–2022, 2017–2022). Note: A blue line depicts the year when an article was published, and a red segment corresponds to the duration of a citation burst.

**Additional file 4**. **Table S3**: Summary of the largest clusters identified in the co-authorship network (2000–2022).

**Additional file 5**. **Table S4**: Summary of the largest clusters identified in the co-citation network of authors (2017–2022).

**Additional file 6**. **Table S5**: The top countries and institutions, ranked by betweenness centrality and citation counts (2000–2022, 2017–2022).

**Additional file 7**. **Table S6**: Average citations and total link strength of countries, institutions, journals, references, and authors per cluster based on bibliographic coupling analysis.

## Data Availability

Not applicable.
