# Supplementary material for: A 2-decade bibliometric analysis of epigenetics of cardiovascular disease: from past to present
Source: Clin Epigenetics. 2023 Nov 25;15:184. doi: 10.1186/s13148-023-01603-9 (PMC10676610; doi:10.1186/s13148-023-01603-9)
Supplement: Supplementary file 1 — Additional file 1. Figure S1: Flow chart of the bibliometric study. Figure S2: Detailed information on the most important clusters of the co-citation network of references ranked by citation bursts for the period 2000–2022. For each cluster, we listed the top five keywords and labeled the cluster with the most-cited keyword (generated by comparing the likelihood ratio of keywords). These keywords are highly predictive of the overall topic of a cluster. The citation burst of each cluster is represented by the tree rings surrounding the nodes. Figure S3: Link walkthrough between clusters based on citation bursts for the co-citation network of references (2000–2022). Figure S4: Co-citation network of references (A), corresponding clusters (B), and timeline visualization of the network (C) for the period 2017–2022. Figure S5: Detailed information on all 17 extracted clusters of the co-citation network of references ranked by citation bursts for the period 2017–2022. Figure S6: Co-citation network of references (A), corresponding clusters (B), and timeline visualization of the network (C) for the year 2022. Figure S7: Overlay visualization of the co-occurring network of keywords (2000–2022) (A) with scores based on the average publication year (B). Note: The minimum number of keyword co-occurrences should exceed 51,000. Each node represents a co-occurring keyword, with the size of the node being proportional to the frequency of keyword co-occurrence and the color of the node varying from blue to yellow depending on the average publication year of articles incorporating this keyword (keywords occurring in earlier years were colored in blue, and those appearing later were colored in yellow). The co-occurrence network is weighted on total link strength across different nodes, which is scored based on the average publication year. Figure S8: Annual scientific production (A) and average citation per year for references (B) (2000–2022). Figure S9: The top 10 growth sources of [file 13148_2023_1603_MOESM1_ESM.pdf]

Global advances in epigenetics of cardiovascular disease: past, present, and future

Yukang Mao[1,2#], Kun Zhao[2#], Nannan Chen[3], Qiangqiang Fu[4], Yimeng Zhou[3], Chuiyu Kong[5,6*], Peng Li[2,7*], Chuanxi Yang[3*]

[1]Department of Cardiology, The Affiliated Suzhou Hospital of Nanjing Medical University, Suzhou Municipal Hospital, Gusu School, Nanjing Medical University, Suzhou, Jiangsu, 215000, China.

[2]Department of Cardiology, The First Affiliated Hospital of Nanjing Medical University, No. 300 Guangzhou Road, Nanjing, Jiangsu, 210029, China.

[3]Department of Cardiology, Yangpu Hospital, Tongji University School of Medicine, Shanghai, 200090, China.

[4]Department of General Practice, Clinical Research Center for General Practice, Yangpu Hospital, Tongji University School of Medicine, Shanghai, 200090, China.

[5]Department of Cardio-Thoracic Surgery, Nanjing Drum Tower Hospital, The Affiliated Hospital of Nanjing University Medical School, Nanjing University, No. 321 Zhongshan Rd, Nanjing, Jiangsu, China.

[6]Institute of Cardiothoracic Vascular Disease, Nanjing University, Nanjing, China.

[7]Key Laboratory of Targeted Intervention of Cardiovascular Disease, Collaborative Innovation Center for Cardiovascular Disease Translational Medicine, Nanjing Medical University. 300 Guangzhou Road, Nanjing, Jiangsu, 210029, China.

#These authors contributed equally to this work.

* Correspondence Author:

Chuanxi Yang, Department of Cardiology, Yangpu Hospital, Tongji University School of Medicine, 450 Tengyue Road, Shanghai, 200090, China. Email: 2205515@tongji.edu.cn

Chuiyu Kong, Department of Cardio-Thoracic Surgery, Nanjing Drum Tower Hospital, The Affiliated Hospital of Nanjing University Medical School, No. 321 Zhongshan Rd, Nanjing, 210008, Jiangsu, China. E-mail: cykong@njmu.edu.cn

Peng Li, Department of Cardiology, the First Affiliated Hospital of Nanjing Medical University, 300 Guangzhou Road, Nanjing 210029, Jiangsu, China. E-mail: lipeng198610@163.com

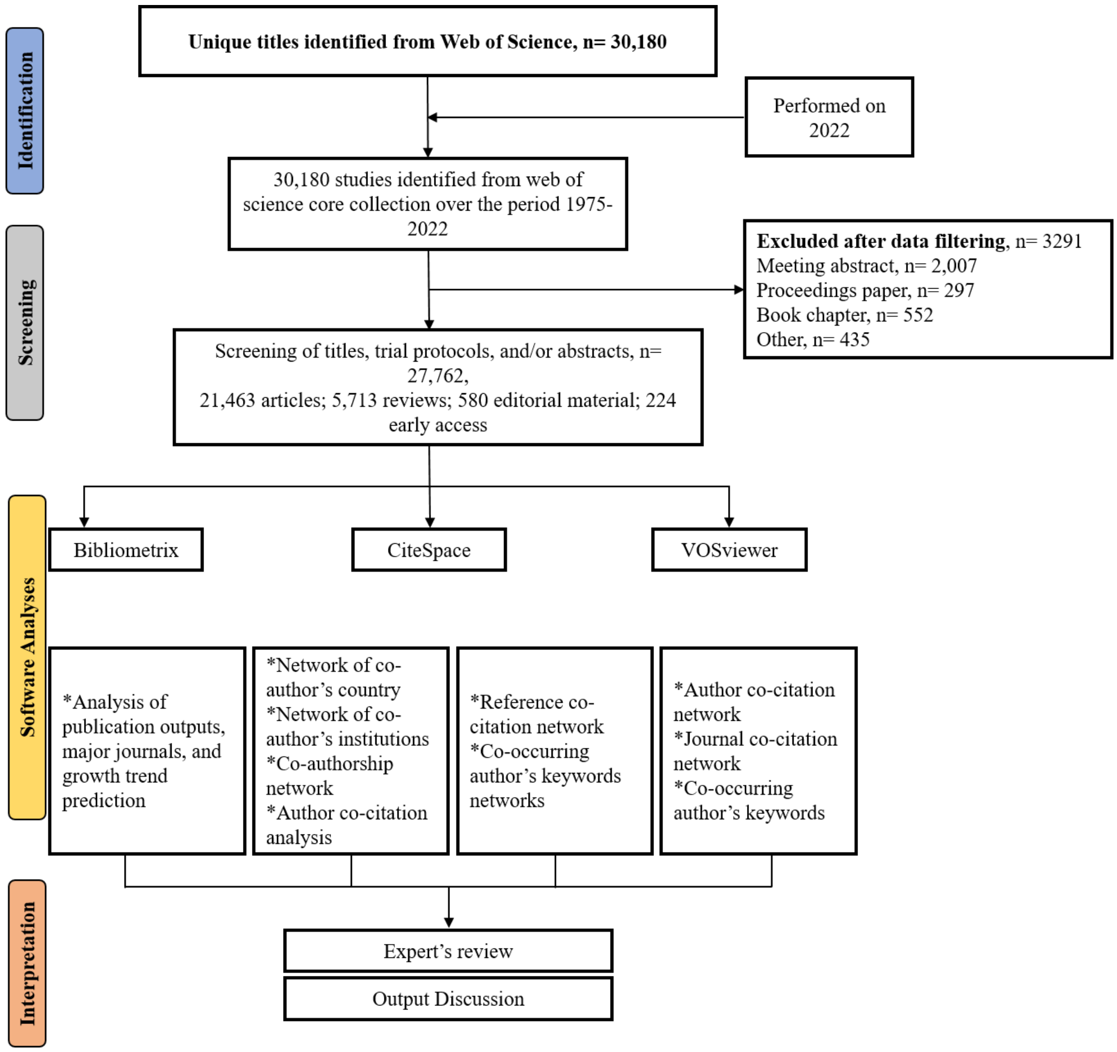

Supplementary Fig. S1. Flow chart of the bibliometric study.

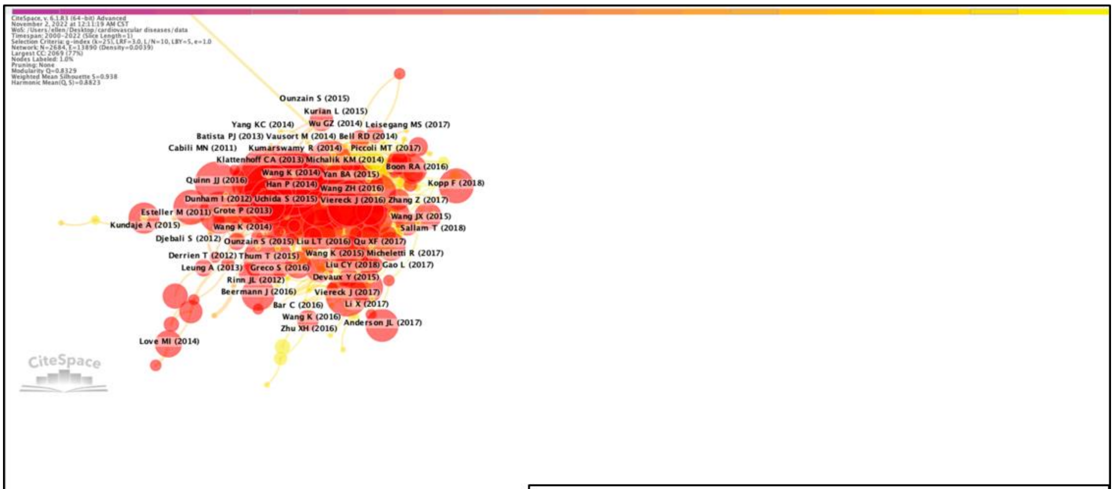

Cluster 0- 'lncrna': lncrna (234.35, 1.0E-4); long noncoding rna (174.37, 1.0E-4); long non-coding rna (131.21, 1.0E-4); microrna (69.41, 1.0E-4); long noncoding rnas (67.63, 1.0E-4)

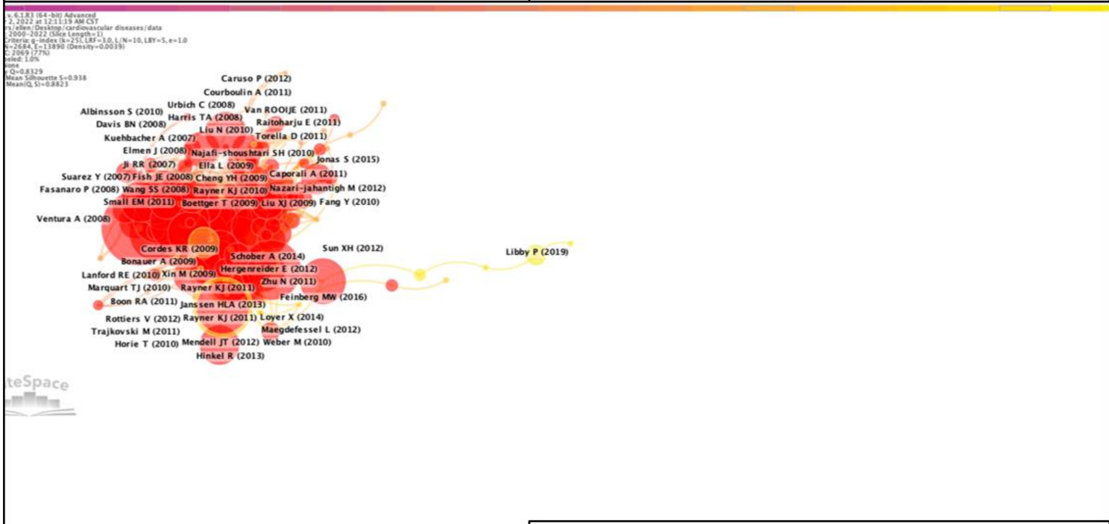

Cluster 1- 'atherosclerosis': atherosclerosis (87.9, 1.0E-4); microrna (79.29, 1.0E-4); micrornas (44.94, 1.0E-4); angiogenesis (35.24, 1.0E-4); abca1 (33.72, 1.0E-4)

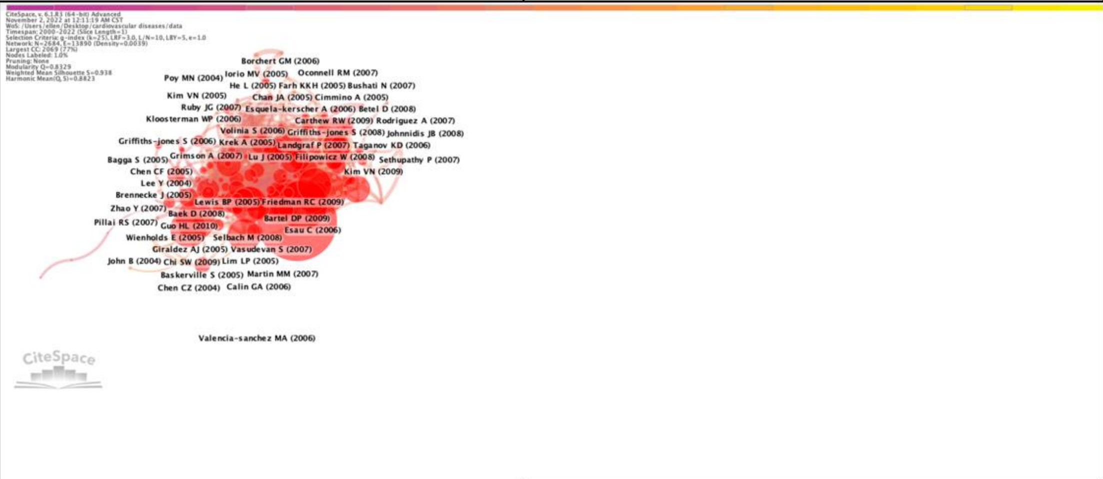

Cluster 2- 'microrna': microrna (48.31, 1.0E-4); gene silencing (25.01, 1.0E-4); dicer (24.96, 1.0E-4); exosomes (19.07, 1.0E-4); immunity (19.05, 1.0E-4)

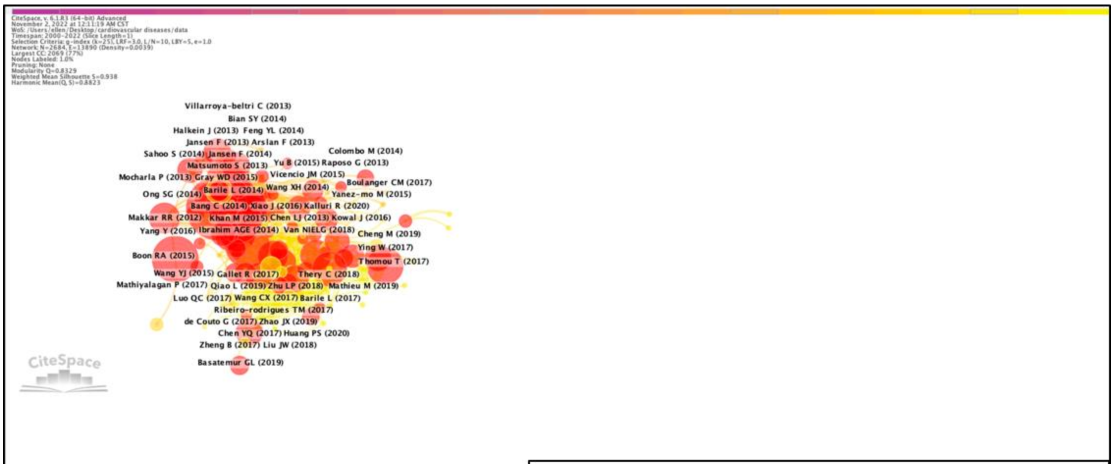

Cluster 3- 'exosomes': exosomes (579.12, 1.0E-4); extracellular vesicles (390.33, 1.0E-4); exosome (167.95, 1.0E-4); mesenchymal stem cells (68.49, 1.0E-4); microvesicles (62.03, 1.0E-4)

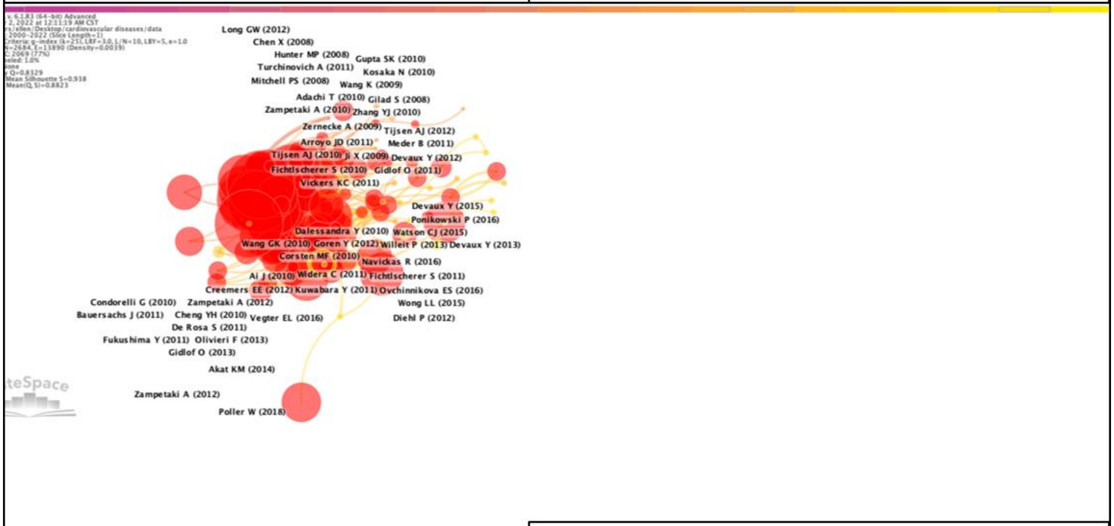

Cluster 4- 'biomarker': biomarker (136.6, 1.0E-4); biomarkers (84.05, 1.0E-4); circulating microrna (53.63, 1.0E-4); acute myocardial infarction (48.39, 1.0E-4); microrna (46.67, 1.0E-4)

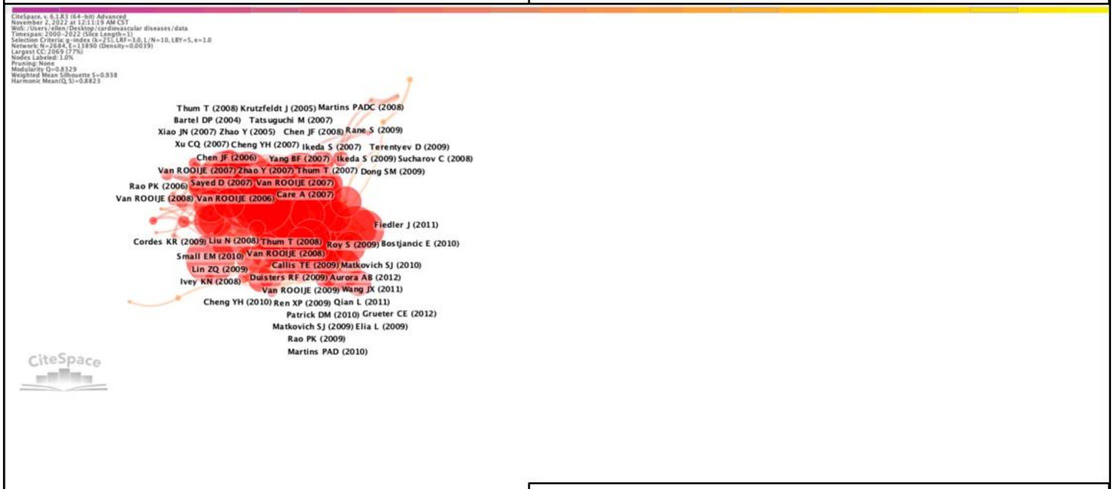

Cluster 5- 'hypertrophy': microrna (62.82, 1.0E-4); exosomes (33.08, 1.0E-4); hypertrophy (31.29, 1.0E-4); cardiac hypertrophy (25.72, 1.0E-4); atherosclerosis (25.57, 1.0E-4)

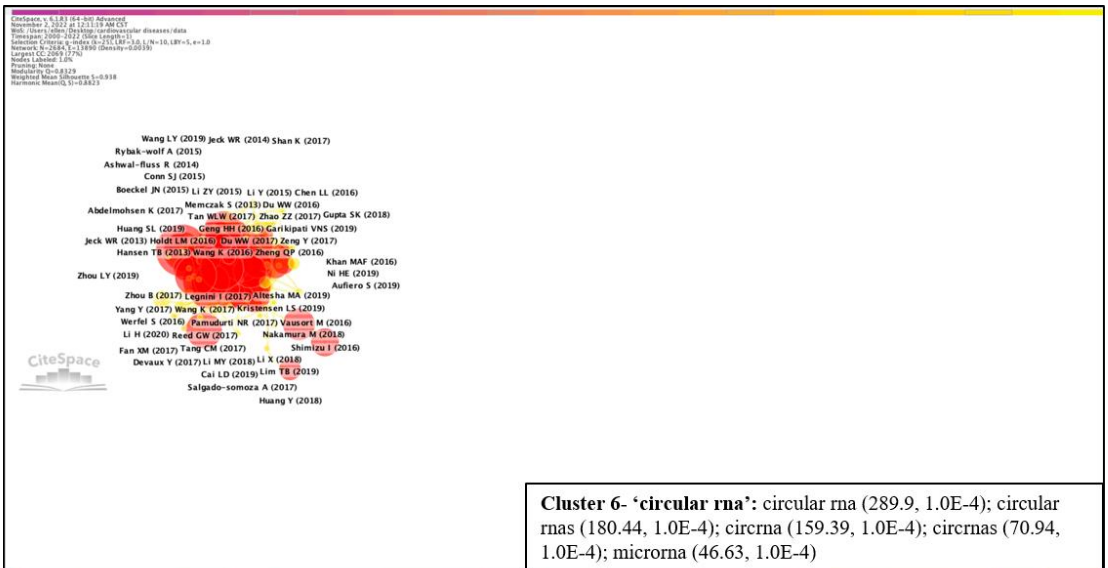

Cluster 6- 'circular rna': circular rna (289.9, 1.0E-4); circular rnas (180.44, 1.0E-4); circrna (159.39, 1.0E-4); circrnas (70.94, 1.0E-4); microrna (46.63, 1.0E-4)

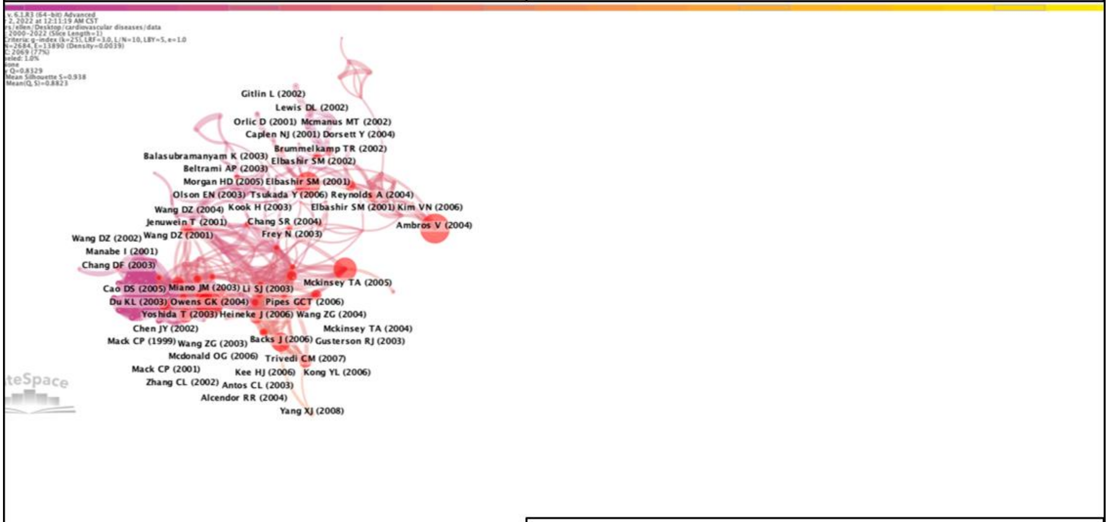

Cluster 7- 'myocardin': myocardin (54.5, 1.0E-4); rna interference (38.66, 1.0E-4); serum response factor (31.95, 1.0E-4); microrna (23.69, 1.0E-4); sirna (23.11, 1.0E-4)

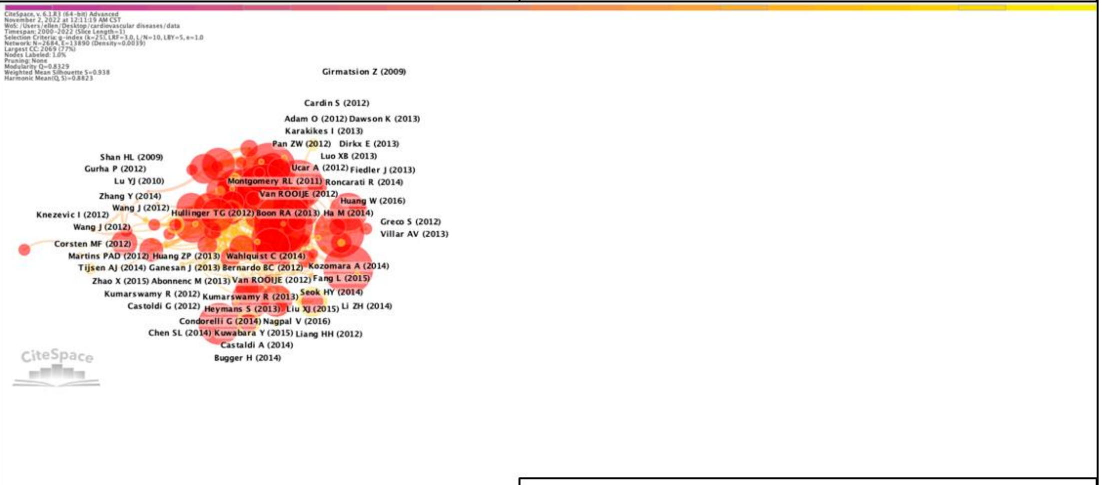

Cluster 8- 'atrial fibrillation': atrial fibrillation (45.94, 1.0E-4); microrna (32.01, 1.0E-4); cardiac fibrosis (26.4, 1.0E-4); exercise training (24.7, 1.0E-4); fibrosis (20.96, 1.0E-4)

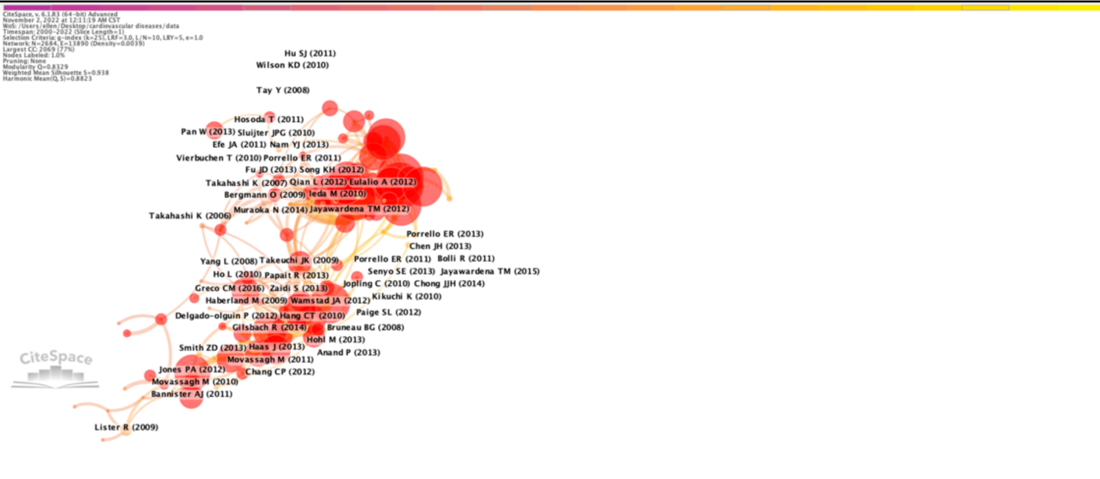

Cluster 9- 'regeneration': regeneration (61.99, 1.0E-4); direct reprogramming (61.94, 1.0E-4); reprogramming (61.7, 1.0E-4); epigenetics (57.27, 1.0E-4); transdifferentiation (56.35, 1.0E-4)

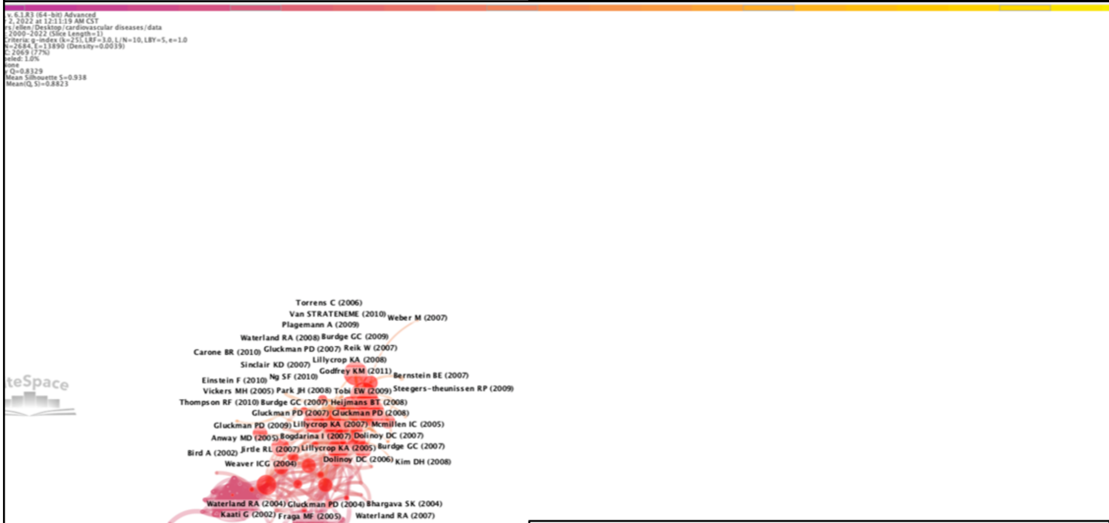

Cluster 10- 'fetal programming': fetal programming (59.91, 1.0E-4); nutrition (41.72, 1.0E-4); epigenetics (39.52, 1.0E-4); microrna (33.02, 1.0E-4); glucocorticoid receptor (24.75, 1.0E-4)

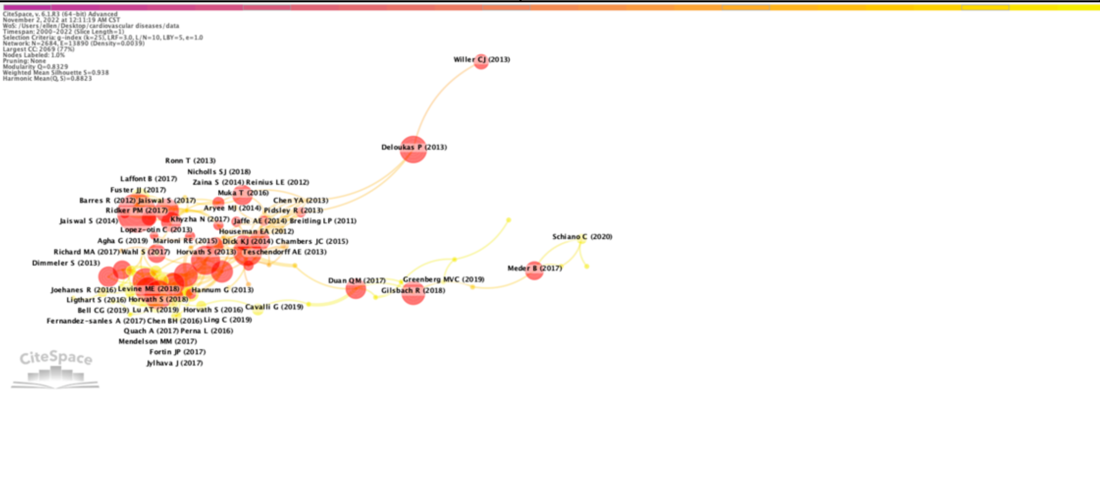

Cluster 11- 'dna methylation': dna methylation (296.76, 1.0E-4); epigenetic clock (115.9, 1.0E-4); aging (113.48, 1.0E-4); microrna (98.18, 1.0E-4); epigenetics (81.26, 1.0E-4)

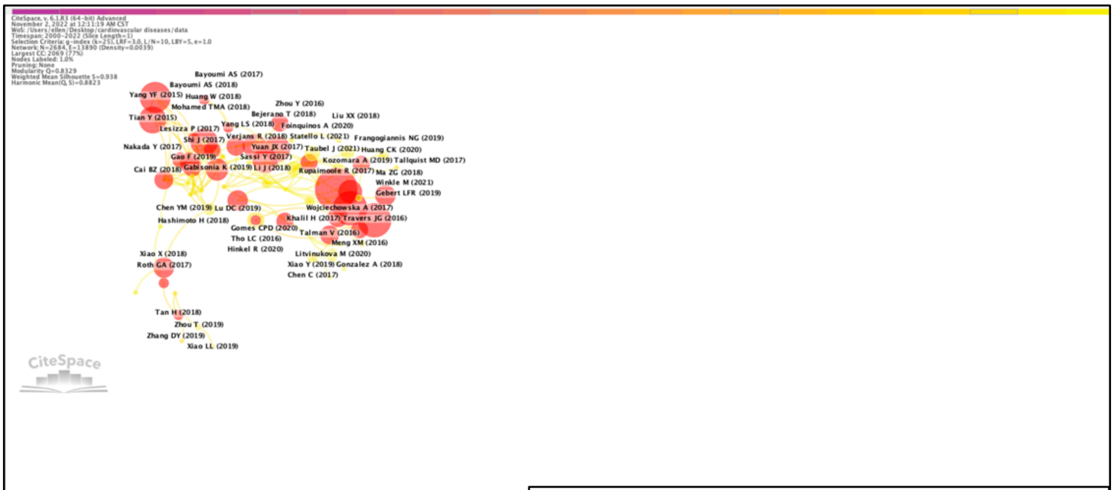

Cluster 12- 'cardiac fibrosis': cardiac fibrosis (53.16, 1.0E-4); fibrosis (29.74, 1.0E-4); myocardial infarction (21.25, 1.0E-4); fibroblast (20.39, 1.0E-4); cardiac fibroblasts (20.39, 1.0E-4)

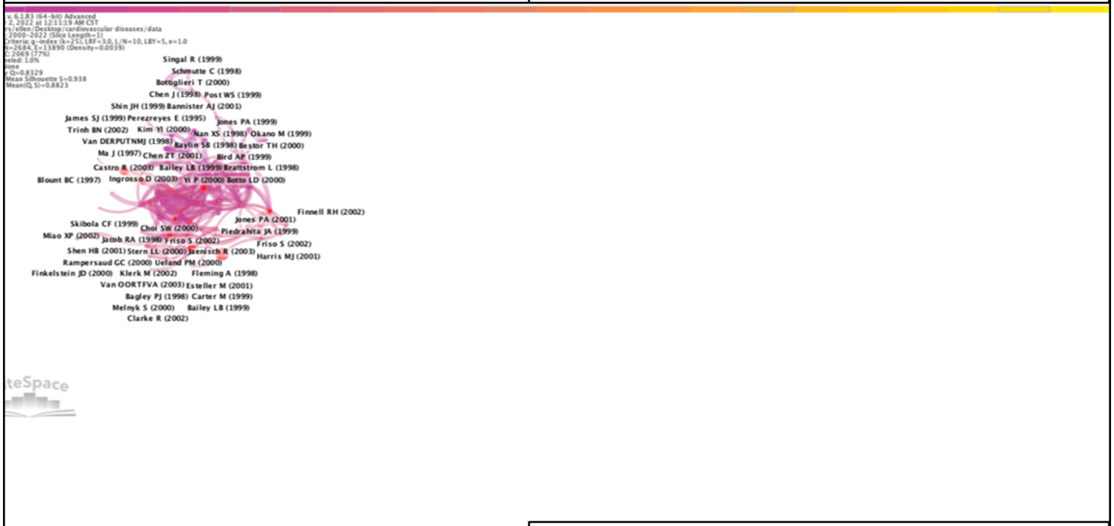

Cluster 13- 'homocysteine': homocysteine (24.07, 1.0E-4); folic acid (20.26, 1.0E-4); folate (20.26, 1.0E-4); thymidylate synthase (12.01, 0.001); 1298a > c (12.01, 0.001)

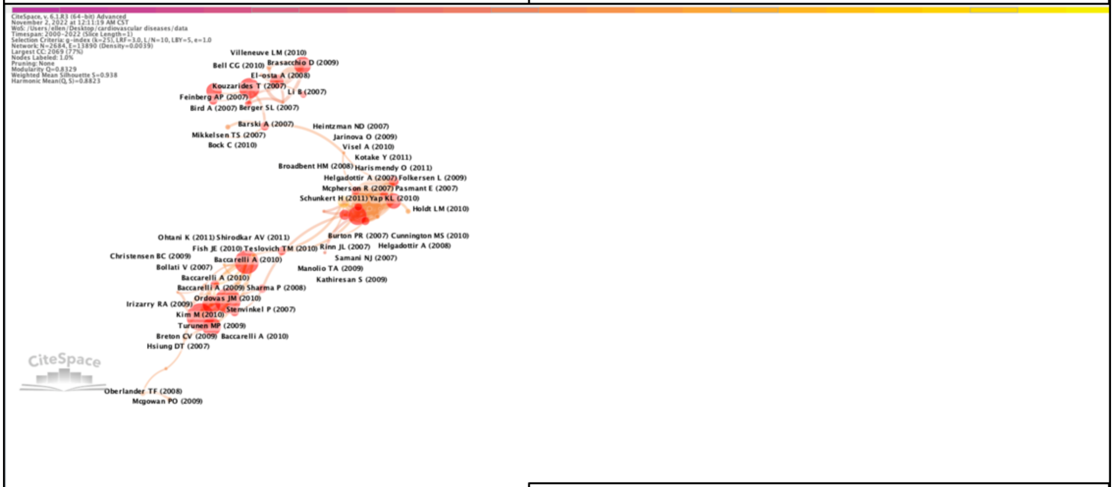

Cluster 14- 'histone modifications': dna methylation (55.34, 1.0E-4); histone modifications (43.91, 1.0E-4); epigenetics (40.68, 1.0E-4); anril (37.19, 1.0E-4); line-1 (37.19, 1.0E-4)

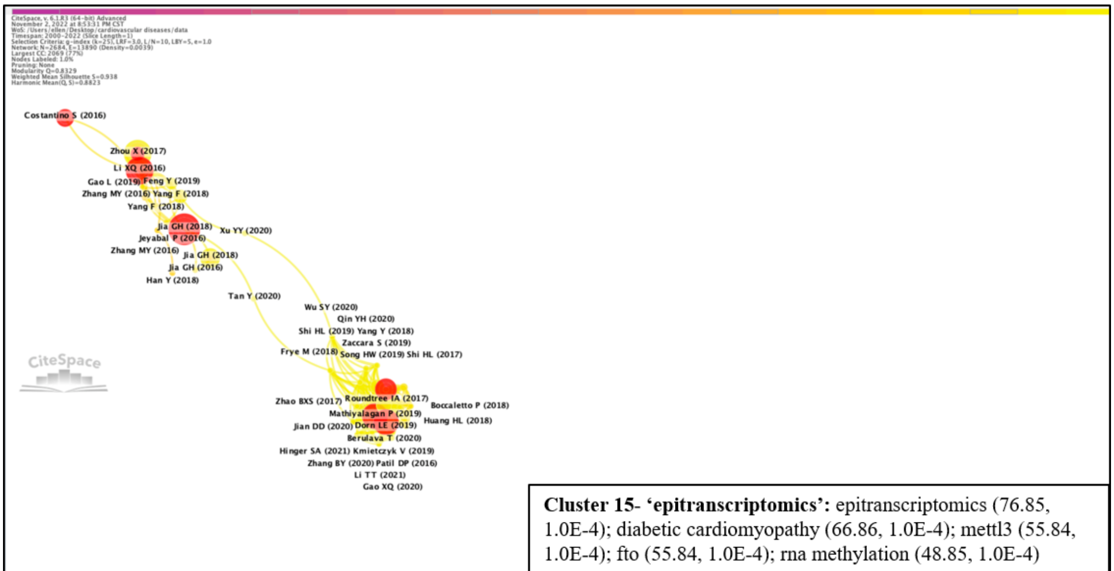

Cluster 15- 'epitranscriptomics': epitranscriptomics (76.85, 1.0E-4); diabetic cardiomyopathy (66.86, 1.0E-4); mettl3 (55.84, 1.0E-4); fto (55.84, 1.0E-4); rna methylation (48.85, 1.0E-4)

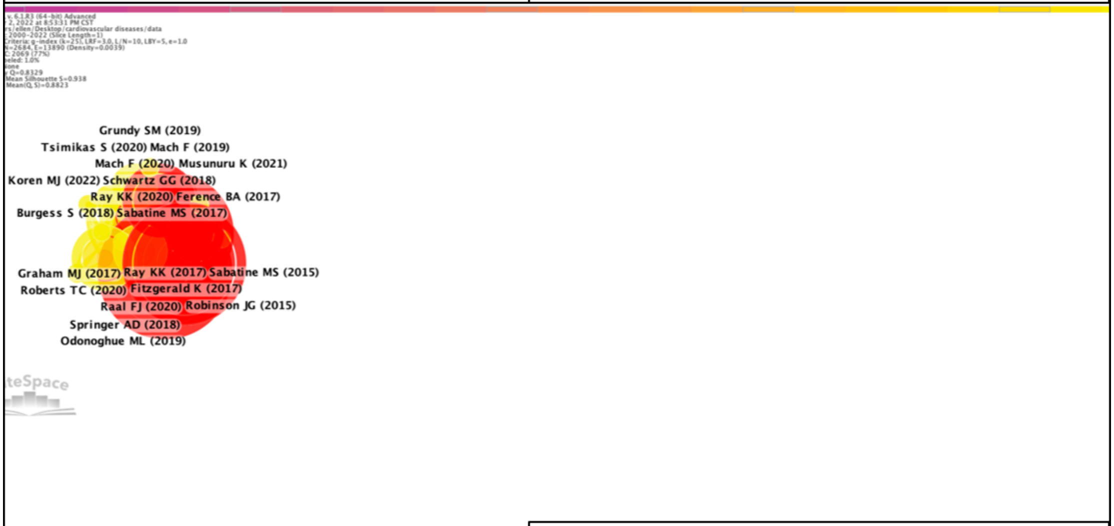

Cluster 16- 'inclisiran': inclisiran (168.61, 1.0E-4); pcsk9 (71.9, 1.0E-4); alirocumab (71.9, 1.0E-4); evolocumab (71.9, 1.0E-4); bempedoic acid (47.88, 1.0E-4)

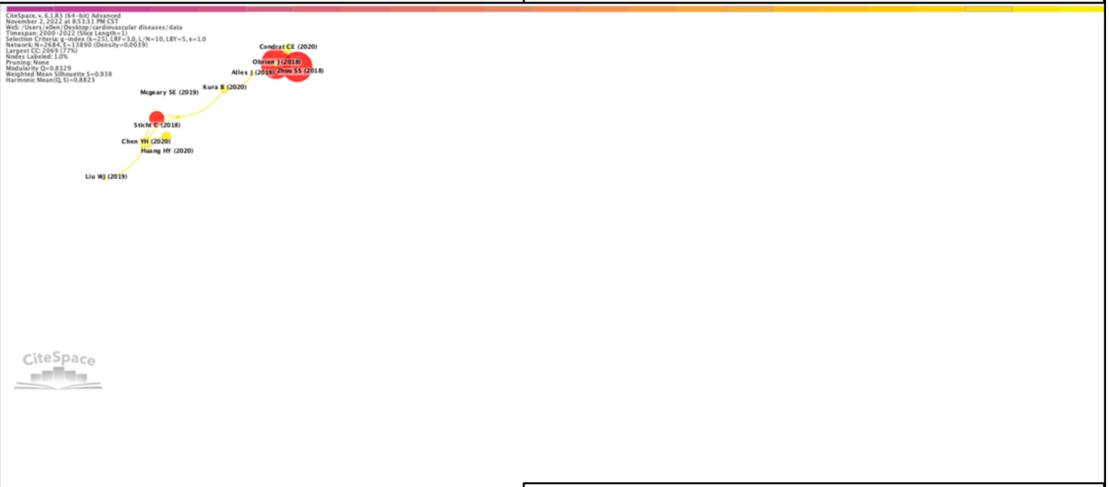

Cluster 17- 'risk factors': risk factors (14.46, 0.001); congenital heart defect (10.7, 0.005); dna methylation (10.19, 0.005); pathogenesis (9.86, 0.005); sepsis (9.25, 0.005)

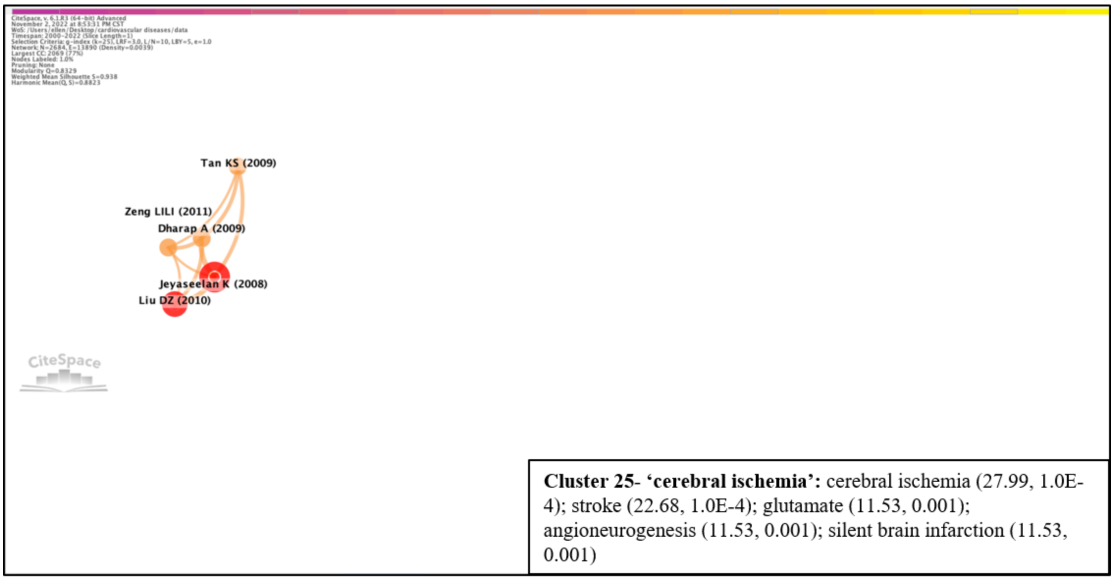

Supplementary Fig. S2. Detailed information on the most important clusters of the co-citation network of references ranked by citation bursts for the time period 2000–2022.

For each cluster, we listed the top five keywords, and labeled the cluster with the most-cited keyword (generated by comparing the likelihood ratio of keywords). These keywords are highly predictive of the overall topic of a cluster. Citation burst of each cluster is represented by the tree rings surrounding the nodes.

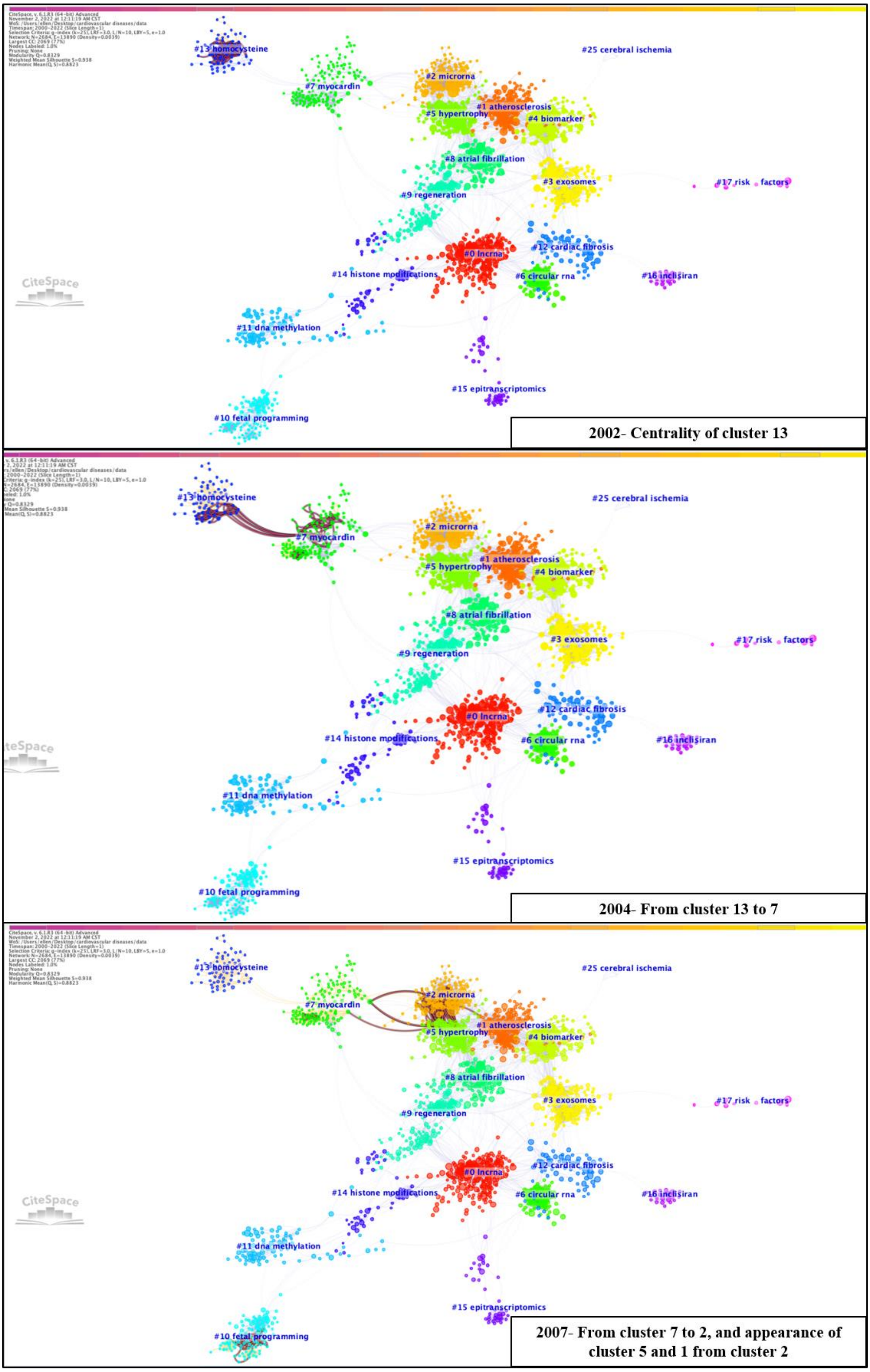

2002- Centrality of cluster 13

2004- From cluster 13 to 7

2007- From cluster 7 to 2, and appearance of cluster 5 and 1 from cluster 2

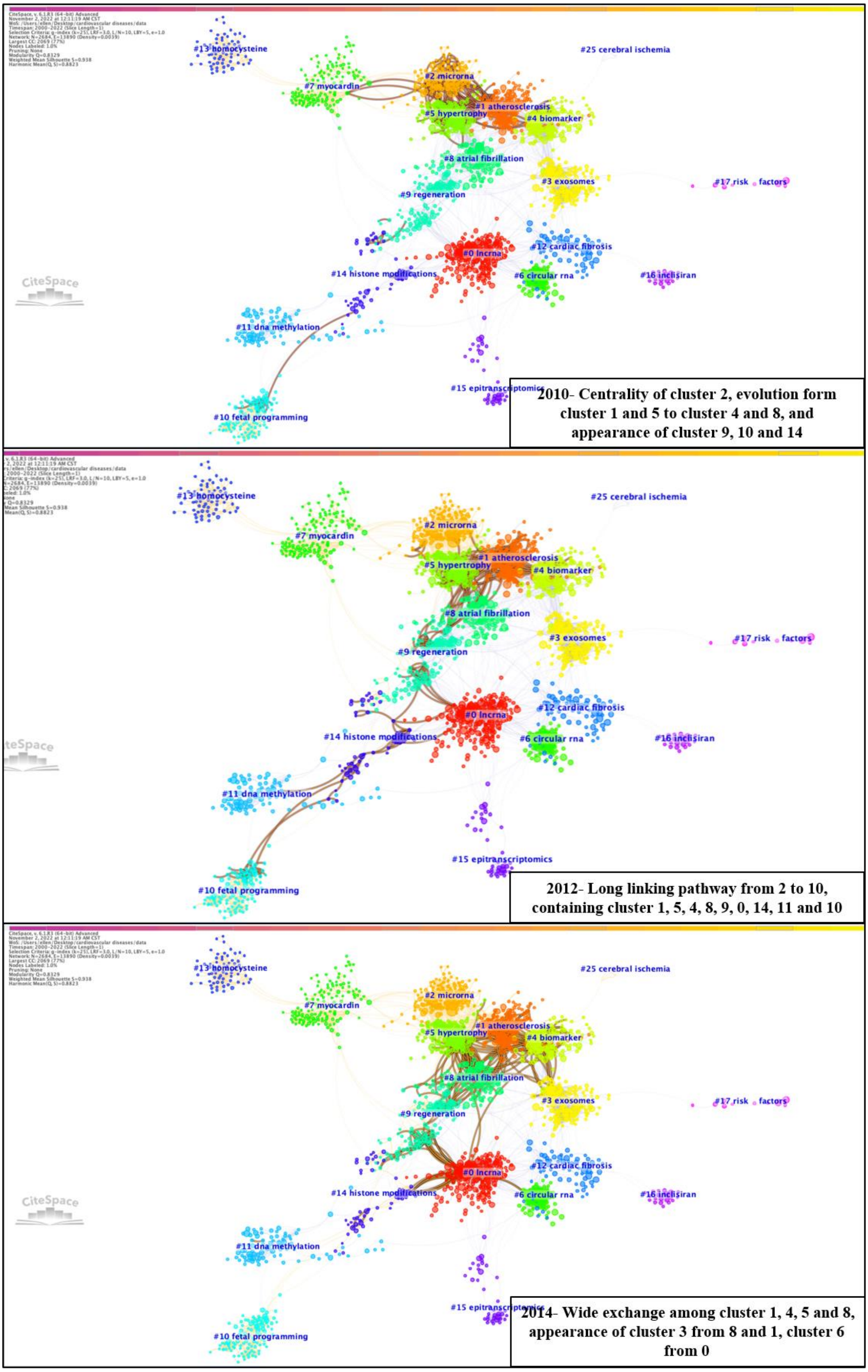
#13 homocysteine
#25 cerebral ischemia
#2 microrna
#7 myocardin
#1 atherosclerosis
#5 hypertrophy
#4 biomarker
#8 atrial fibrillation
#3 exosomes
#17 risk factors
#9 regeneration
#12 cardiac fibrosis
#0 lncrna
#14 histone modifications
#6 circular rna
#16 inclisiran
#11 dna methylation
#15 epitranscriptomics
#10 fetal programming
CiteSpace
2010- Centrality of cluster 2, evolution form cluster 1 and 5 to cluster 4 and 8, and appearance of cluster 9, 10 and 14
2012- Long linking pathway from 2 to 10, containing cluster 1, 5, 4, 8, 9, 0, 14, 11 and 10
2014- Wide exchange among cluster 1, 4, 5 and 8, appearance of cluster 3 from 8 and 1, cluster 6 from 0

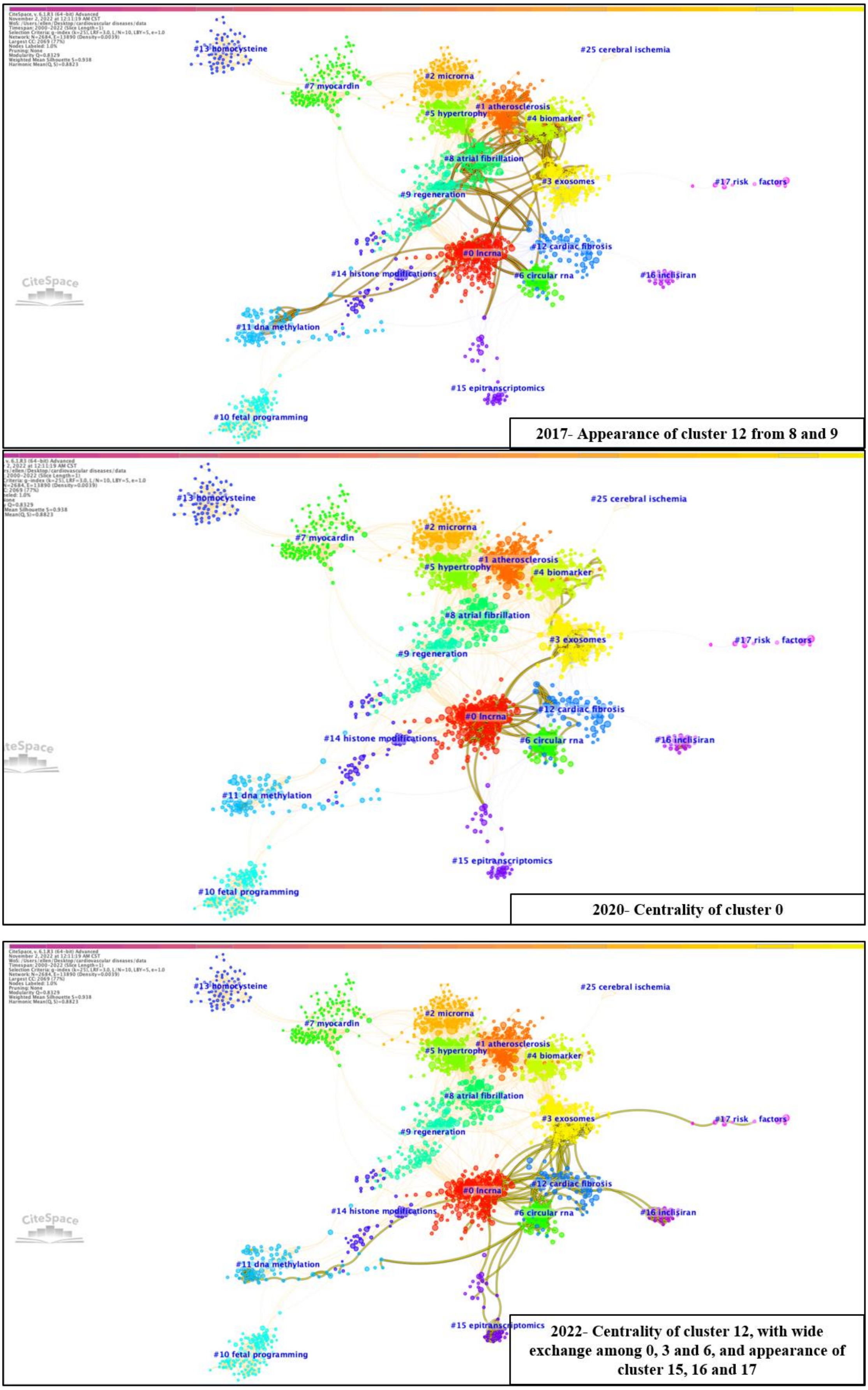

Supplementary Fig. S3. Link walkthrough between clusters based on citation bursts for the co-

citation network of references (2000–2022).

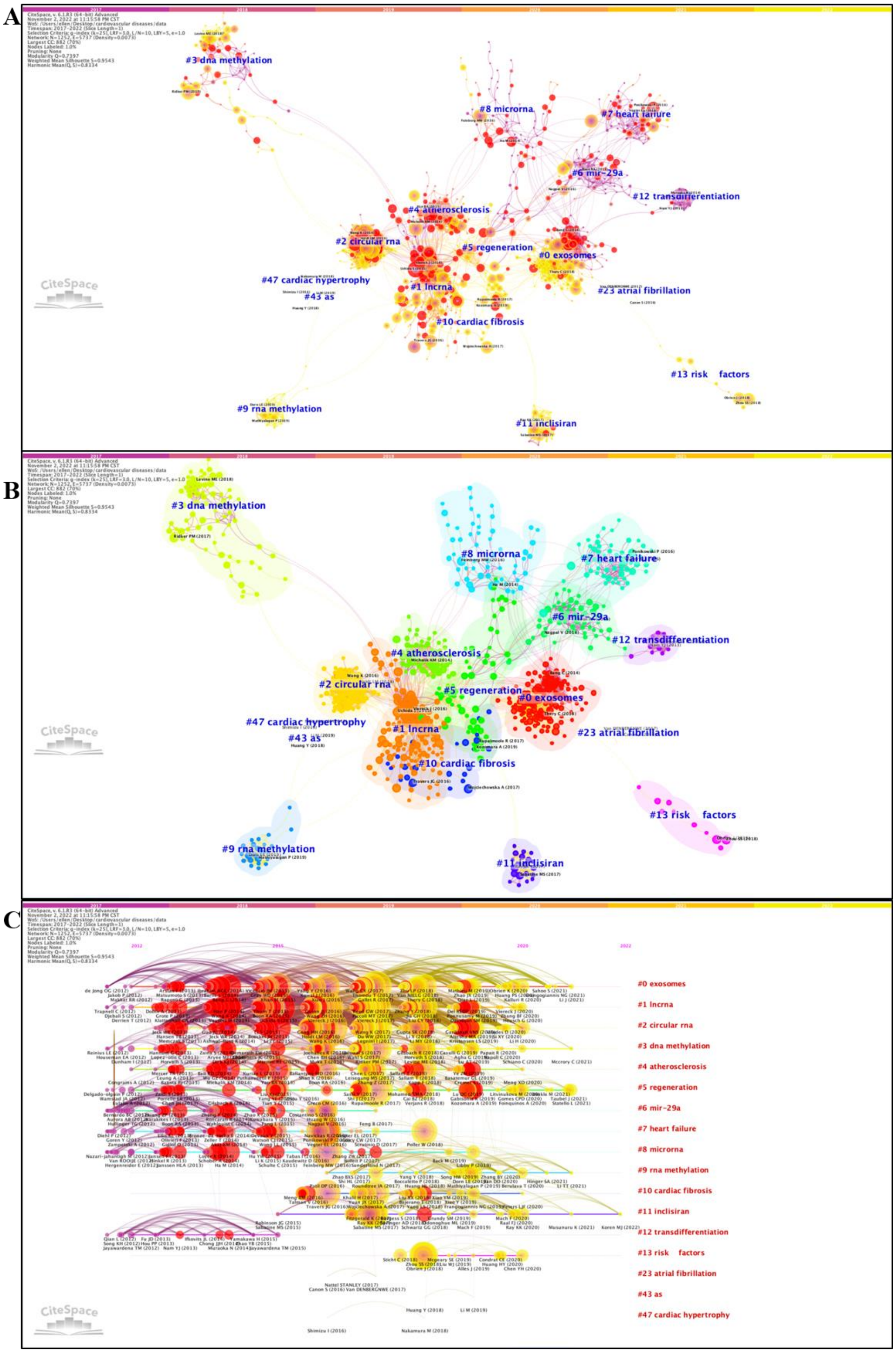

Supplementary Fig. S4. Co-citation network of references (A), corresponding clusters (B), and timeline visualization of the network (C) for the time period 2017–2022.

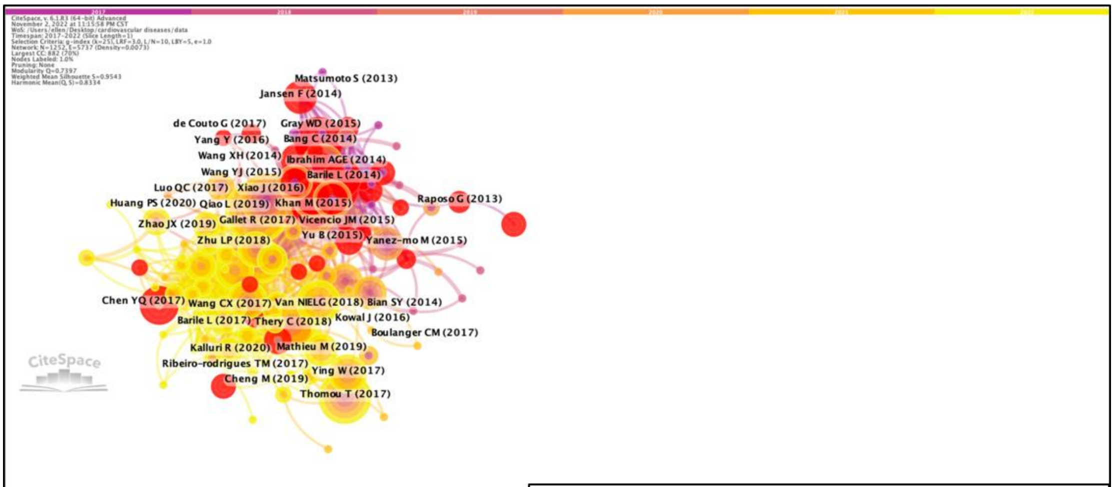

Cluster 0- 'exosomes': exosomes (421.4, 1.0E-4); extracellular vesicles (291.44, 1.0E-4); exosome (95.91, 1.0E-4); microvesicles (57.5, 1.0E-4); stem cells (53.15, 1.0E-4)

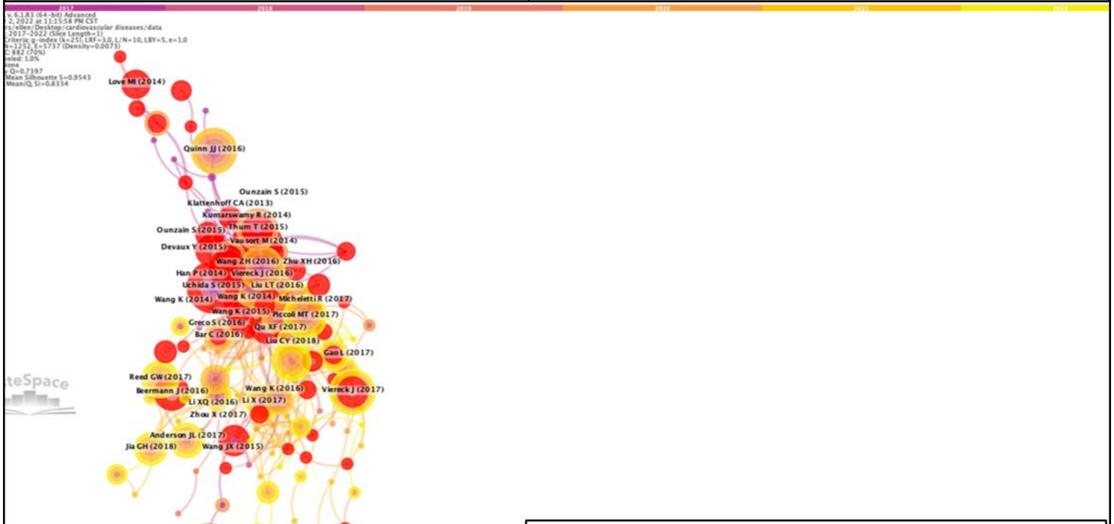

Cluster 1- 'lncrna': lncrna (70.06, 1.0E-4); long non-coding rna (52.16, 1.0E-4); long noncoding rna (49.14, 1.0E-4); apoptosis (44.98, 1.0E-4); exosomes (36.24, 1.0E-4)

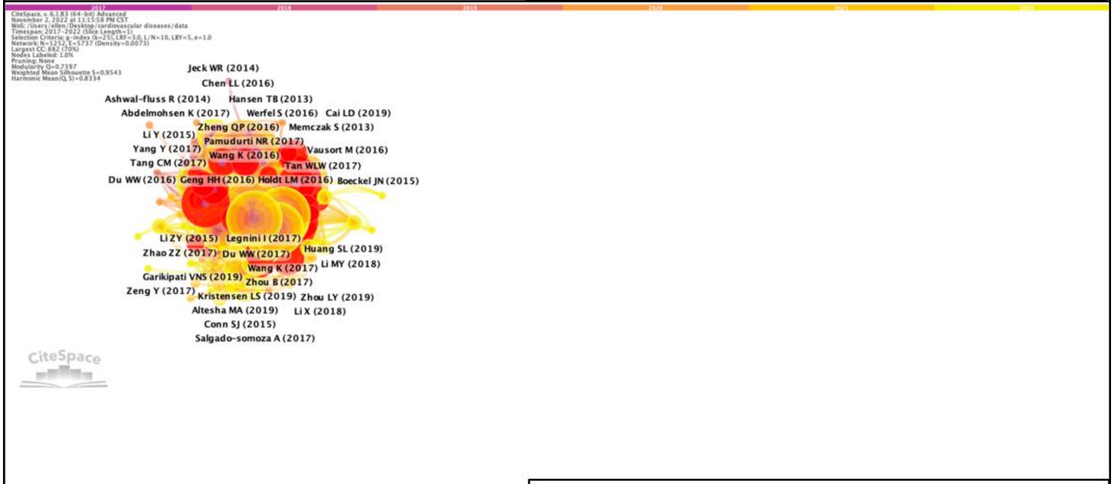

Cluster 2- 'circular rna': circular rna (185.86, 1.0E-4); circular rnas (116.22, 1.0E-4); circrna (81.49, 1.0E-4); circrnas (41.81, 1.0E-4); mirna sponge (40.41, 1.0E-4)

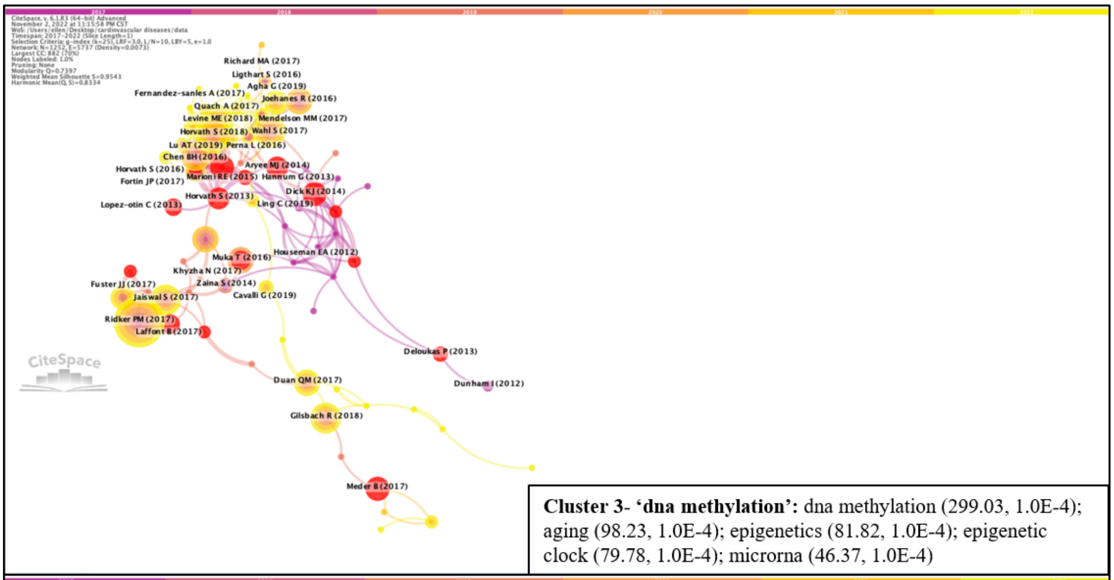

Cluster 3- 'dna methylation': dna methylation (299.03, 1.0E-4); aging (98.23, 1.0E-4); epigenetics (81.82, 1.0E-4); epigenetic clock (79.78, 1.0E-4); microrna (46.37, 1.0E-4)

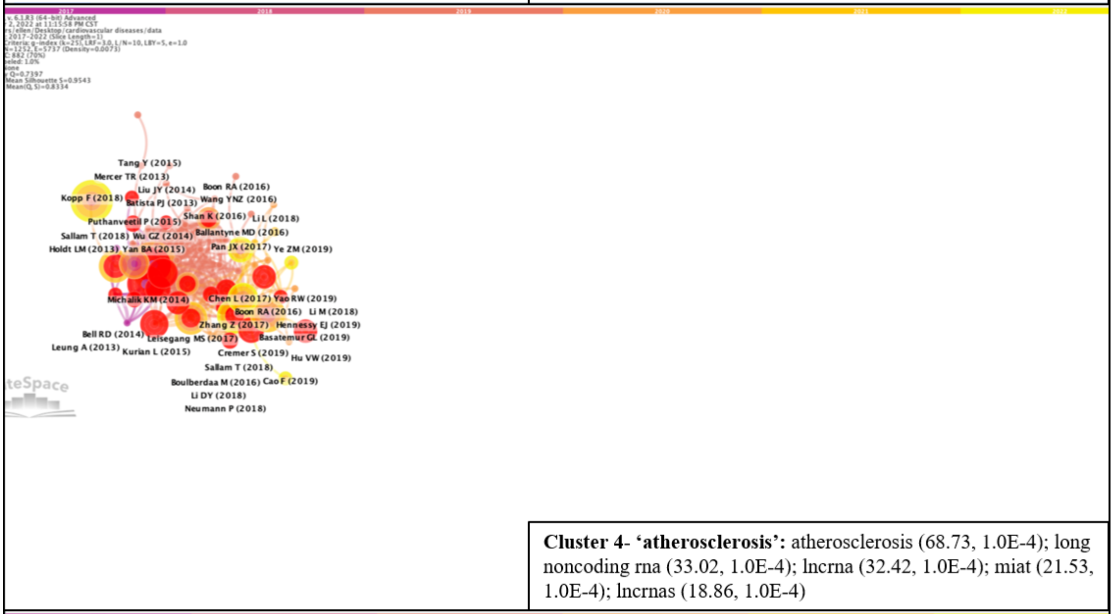

Cluster 4- 'atherosclerosis': atherosclerosis (68.73, 1.0E-4); long noncoding rna (33.02, 1.0E-4); lncrna (32.42, 1.0E-4); miat (21.53, 1.0E-4); lncrnas (18.86, 1.0E-4)

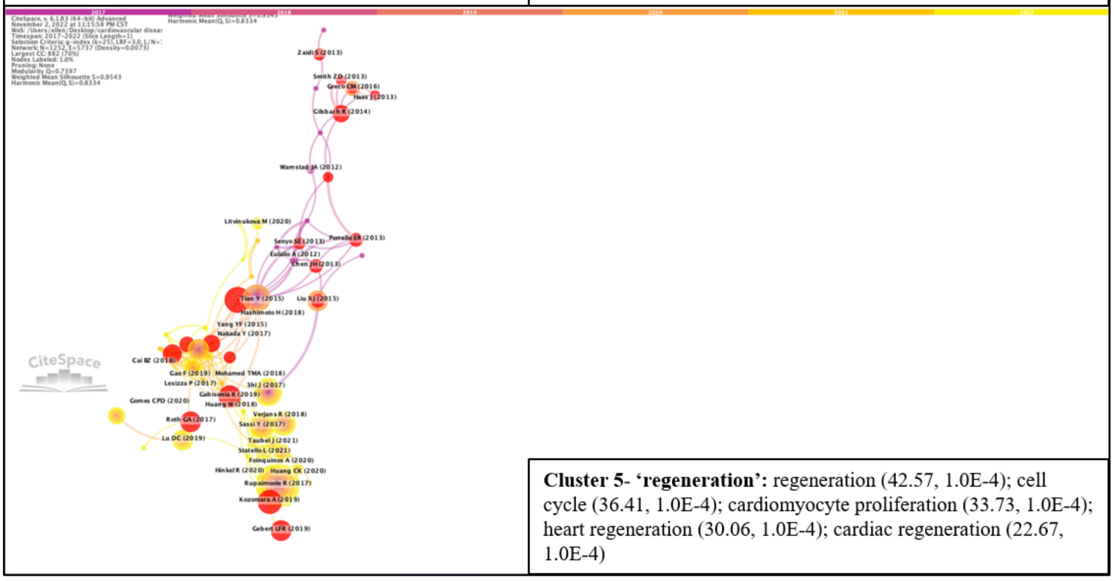

Cluster 5- 'regeneration': regeneration (42.57, 1.0E-4); cell cycle (36.41, 1.0E-4); cardiomyocyte proliferation (33.73, 1.0E-4); heart regeneration (30.06, 1.0E-4); cardiac regeneration (22.67, 1.0E-4)

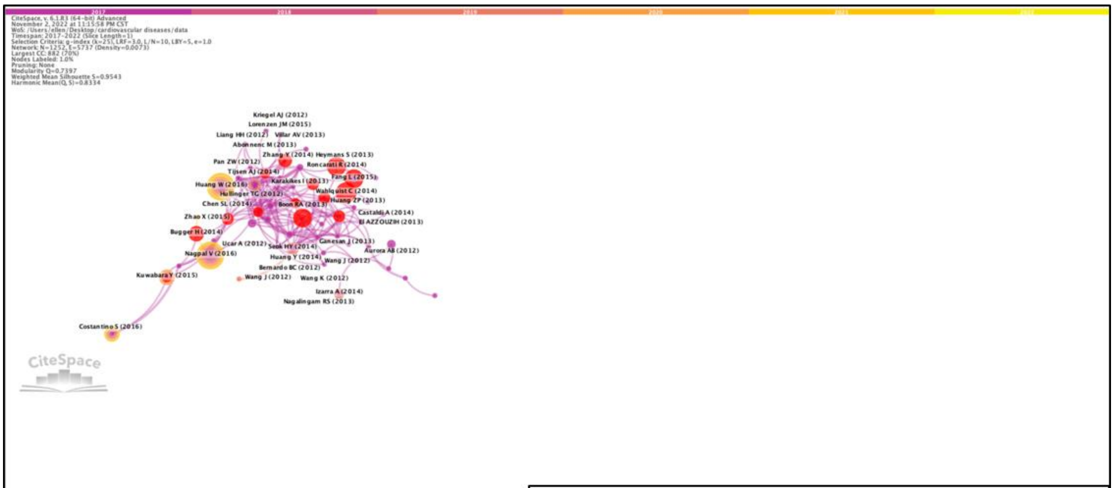

Cluster 6- 'mir-29a': mir-29a (18.9, 1.0E-4); cardiac fibrosis (14.81, 0.001); mir-155 (13.09, 0.001); skeletal muscle (13.09, 0.001); mitochondria (12.67, 0.001)

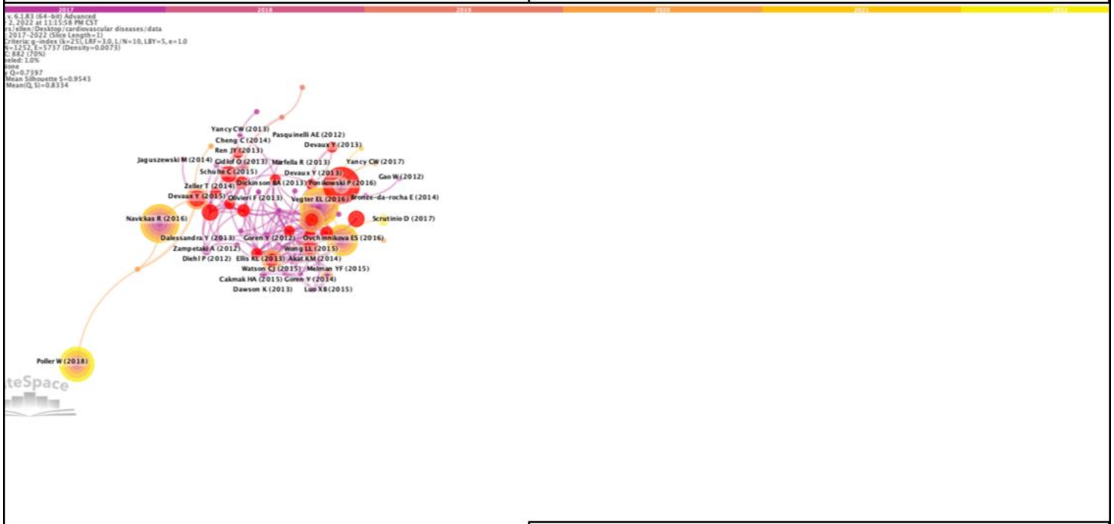

Cluster 7- 'heart failure': heart failure (50.77, 1.0E-4); prognosis (46.85, 1.0E-4); biomarkers (32.46, 1.0E-4); meta-analysis (31.54, 1.0E-4); biomarker (29.03, 1.0E-4)

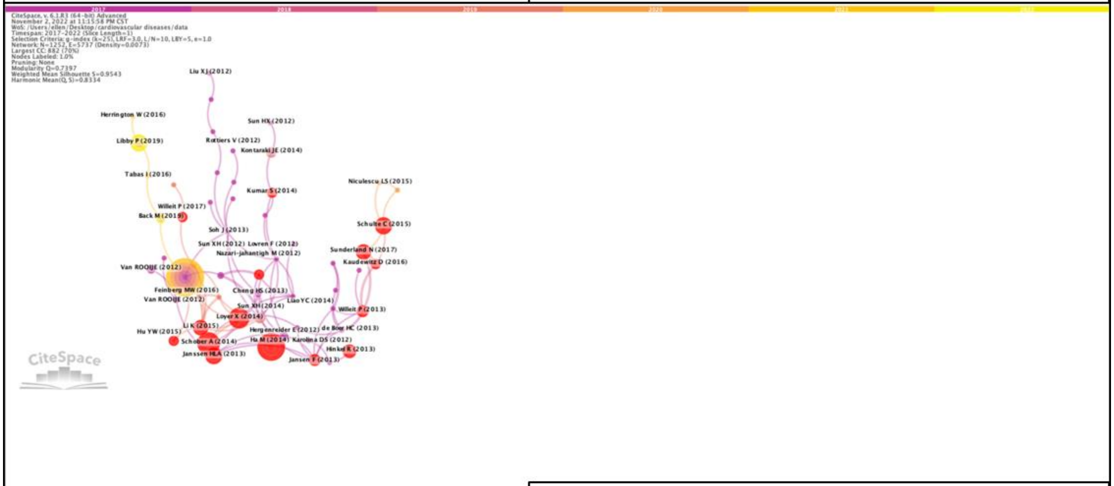

Cluster 8- 'microrna': atherosclerosis (51.98, 1.0E-4); microrna (26.4, 1.0E-4); platelet (21.63, 1.0E-4); restenosis (16.21, 1.0E-4); shear stress (16.21, 1.0E-4)

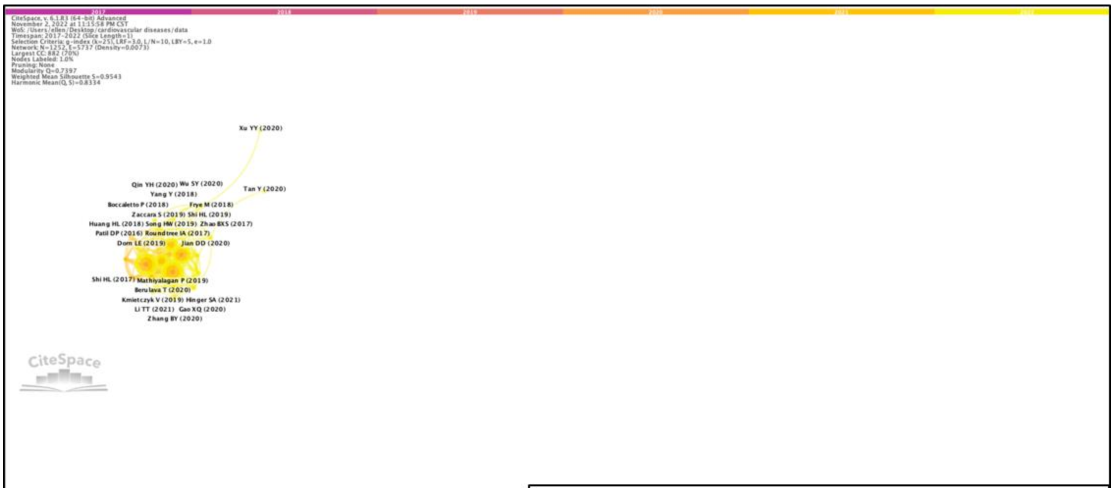

Cluster 9- 'rna methylation': rna methylation (48.26, 1.0E-4); epitranscriptomics (39.33, 1.0E-4); m6a (32.13, 1.0E-4); rna modification (32.13, 1.0E-4); n6-methyladenosine (32.13, 1.0E-4)

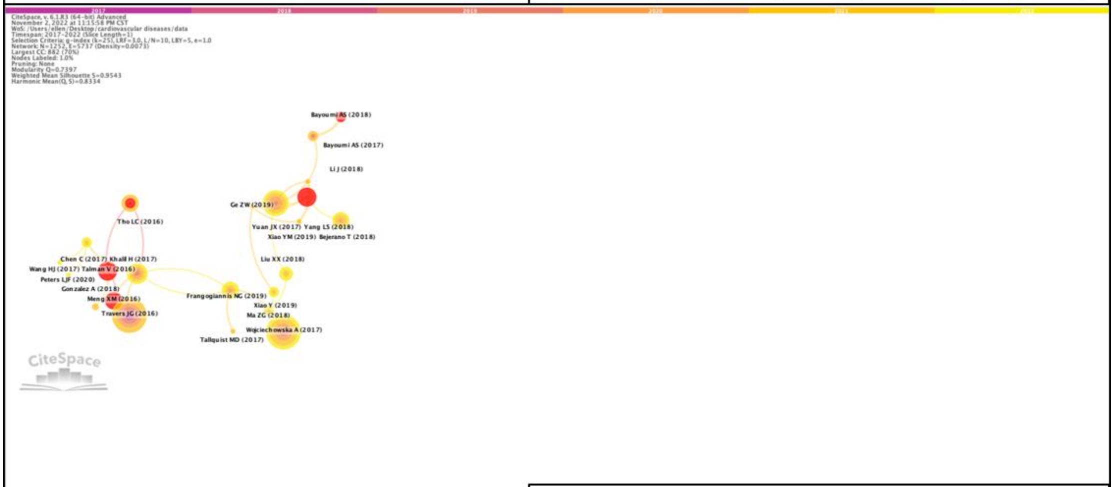

Cluster 10- 'cardiac fibrosis': cardiac fibrosis (86.75, 1.0E-4); cardiac fibroblasts (40.67, 1.0E-4); fibrosis (32.4, 1.0E-4); myocardial fibrosis (17.09, 1.0E-4); fibroblast (13.99, 0.001)

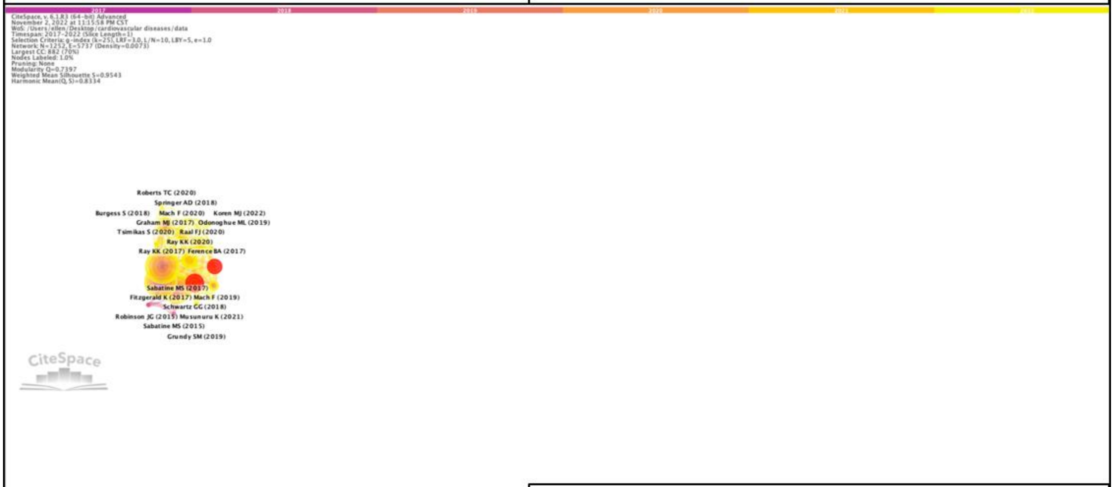

Cluster 11- 'inclisiran': inclisiran (147.69, 1.0E-4); pcsk9 (62.94, 1.0E-4); sirna (62.94, 1.0E-4); alirocumab (62.94, 1.0E-4); evolocumab (62.94, 1.0E-4)

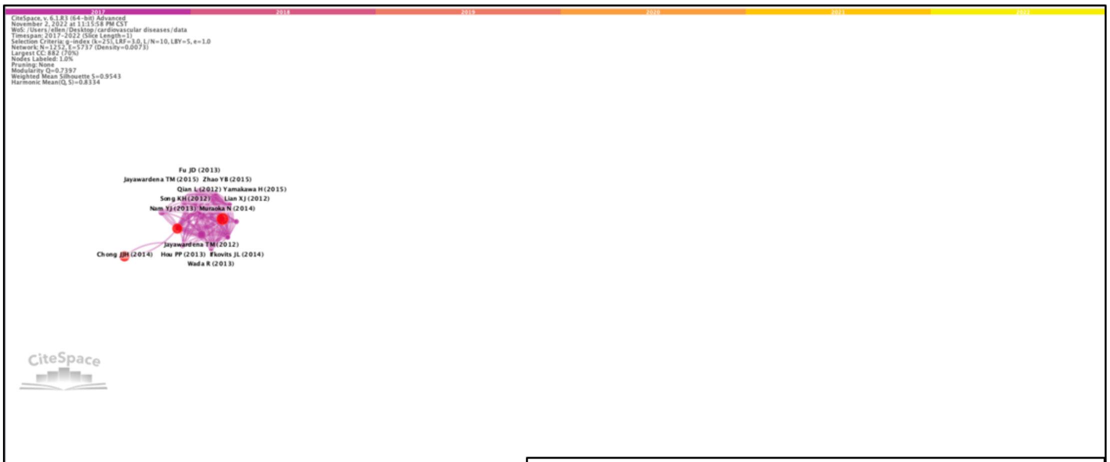

Cluster 12- ‘transdifferentiation’: transdifferentiation (28.7, 1.0E-4); direct cardiac reprogramming (20.44, 1.0E-4); direct reprogramming (19.11, 1.0E-4); cellular reprogramming (15.31, 1.0E-4); transcription factor (13.6, 0.001)

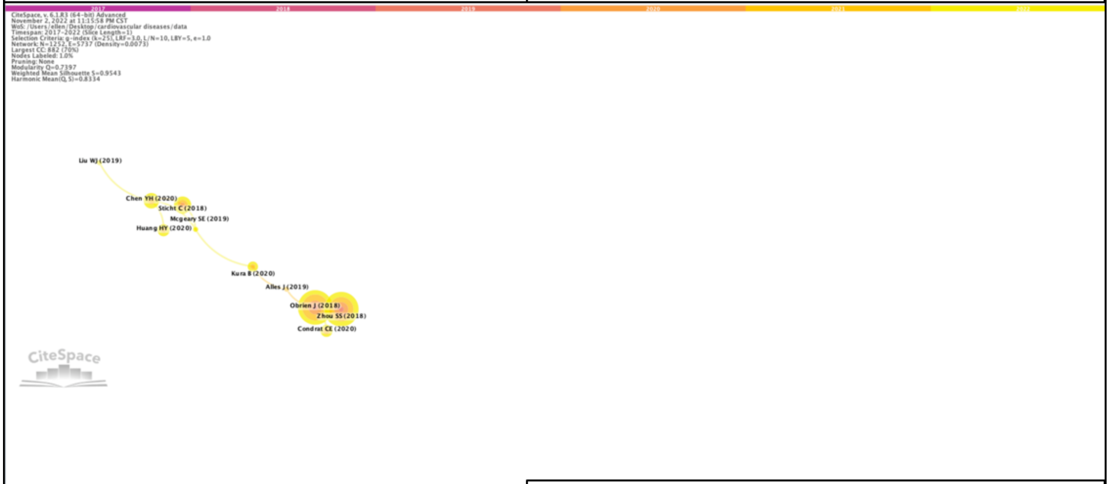

Cluster 13- ‘risk factors’: risk factors (12.47, 0.001); mirna (10.13, 0.005); apoptosis (9.4, 0.005); dna methylation (9.3, 0.005); congenital heart defect (8.74, 0.005)

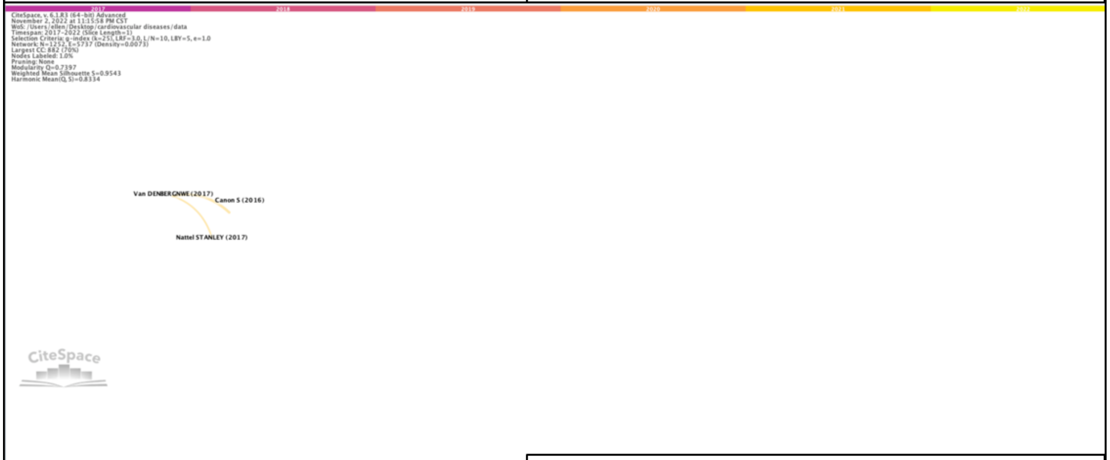

Cluster 23- ‘atrial fibrillation’: atrial fibrillation (41.01, 1.0E-4); laboratory diagnostics (10.57, 0.005); mmps (10.57, 0.005); cardiac autonomic nervous system (10.57, 0.005); microrna-1 (10.57, 0.005)

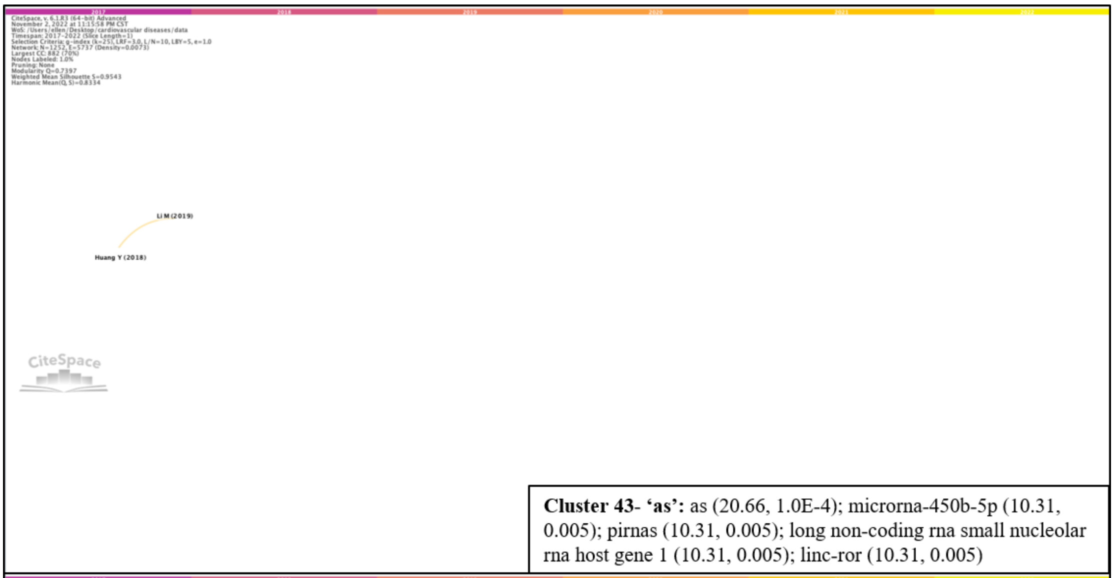

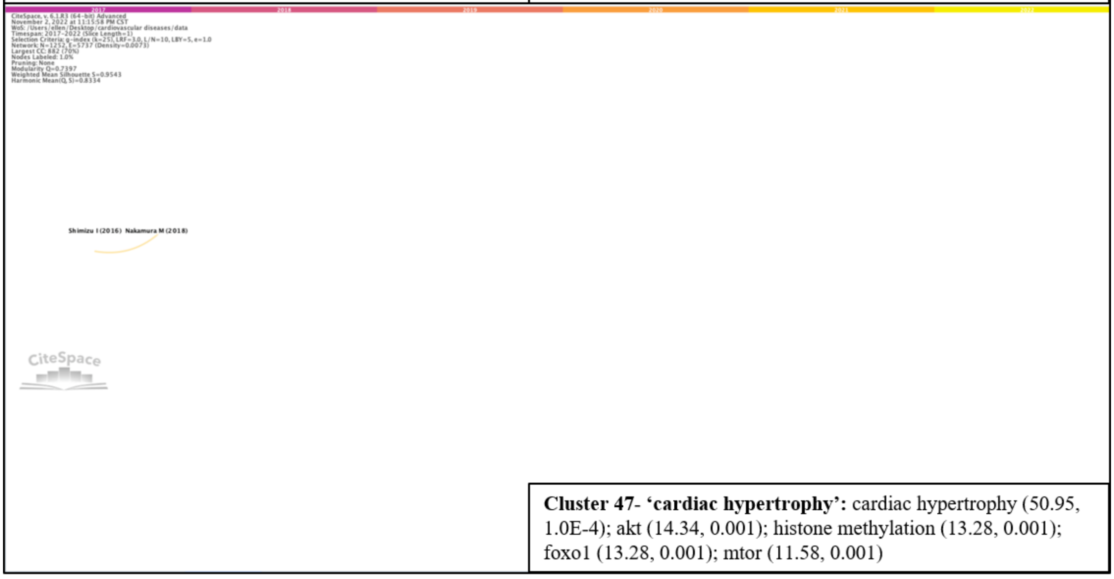

Supplementary Fig. S5. Detailed information on all 17 extracted clusters of the co-citation network of references ranked by citation bursts for the time period 2017–2022.

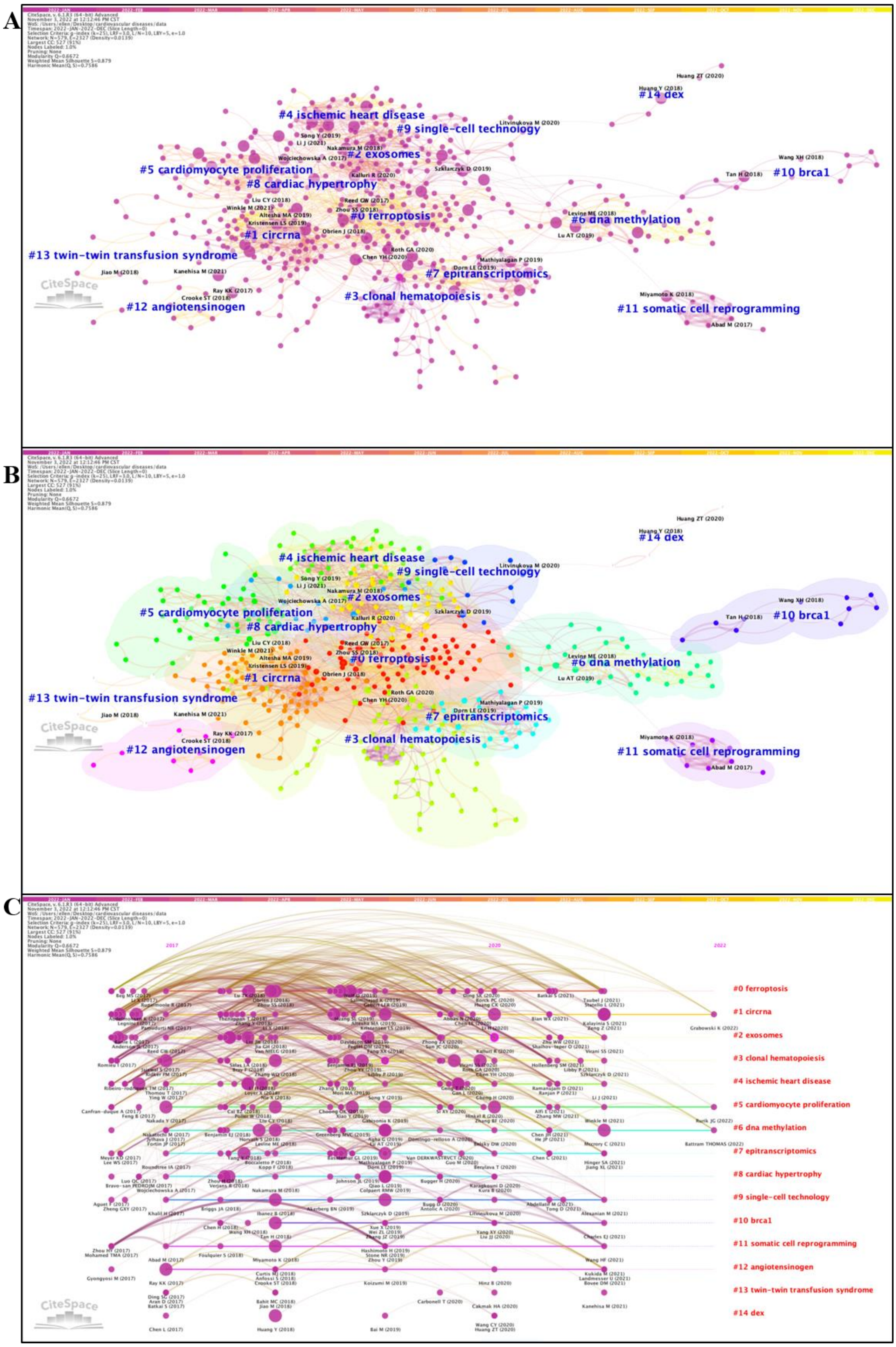

Supplementary Fig. S6. Co-citation network of references (A), corresponding clusters (B), and timeline visualization of the network (C) for the year 2022.

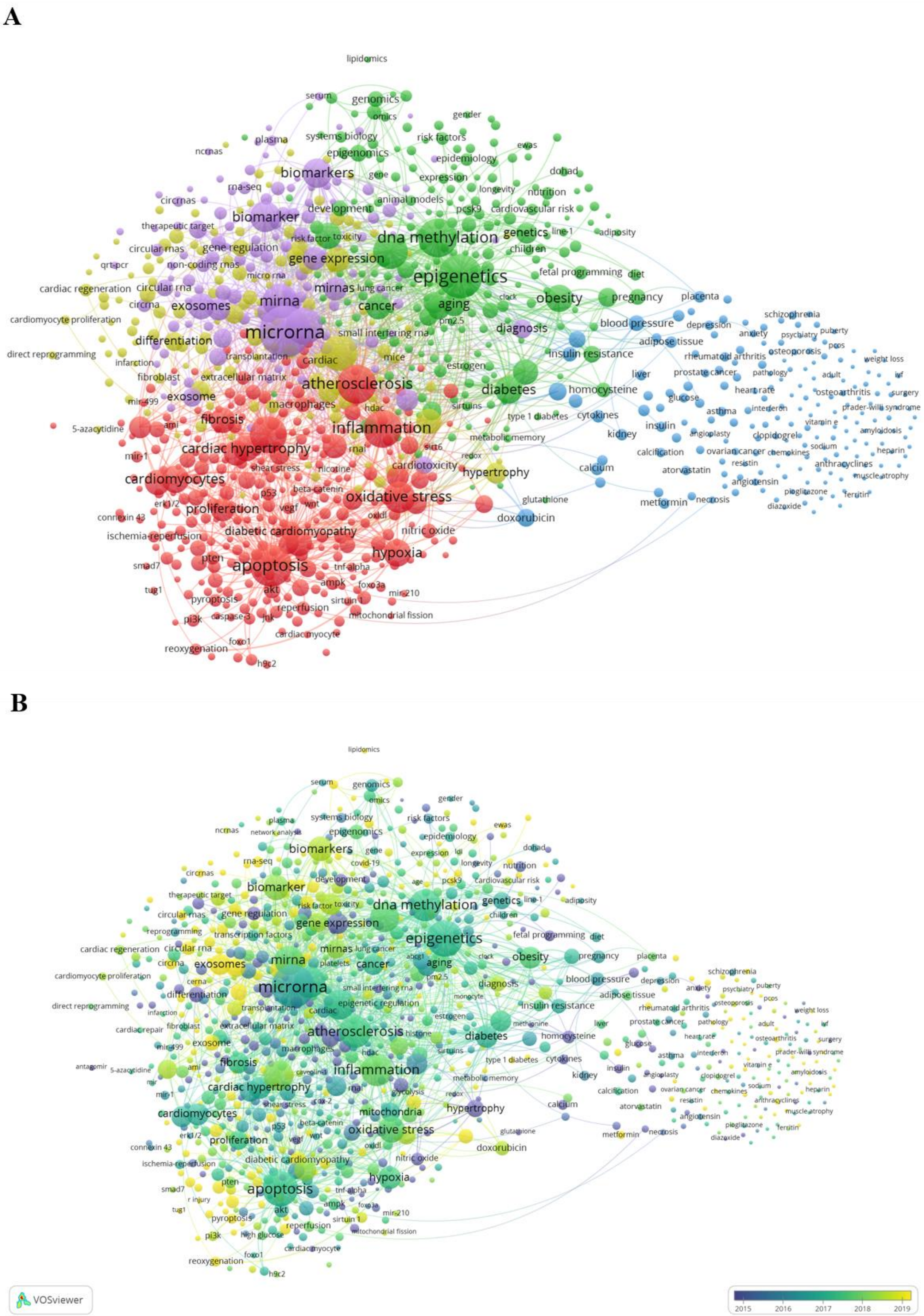

Supplementary Fig. S7. Overlay visualization of the co-occurring network of keywords (2000–2022) (A) with scores based on the average publication year (B).

Note: Minimum number of keyword co-occurrence should exceed 51,000. Each node represents a co-occurring keyword, with the size of the node being proportional to the frequency of keyword co-occurrence and the color of the node varying from blue to yellow depending on the average publication year of articles incorporating this keyword (keywords occurring in earlier years were colored in blue, and

those appearing later were colored in yellow). The co-occurrence network is weighted on total link strength across different nodes, which is scored based on the average publication year.

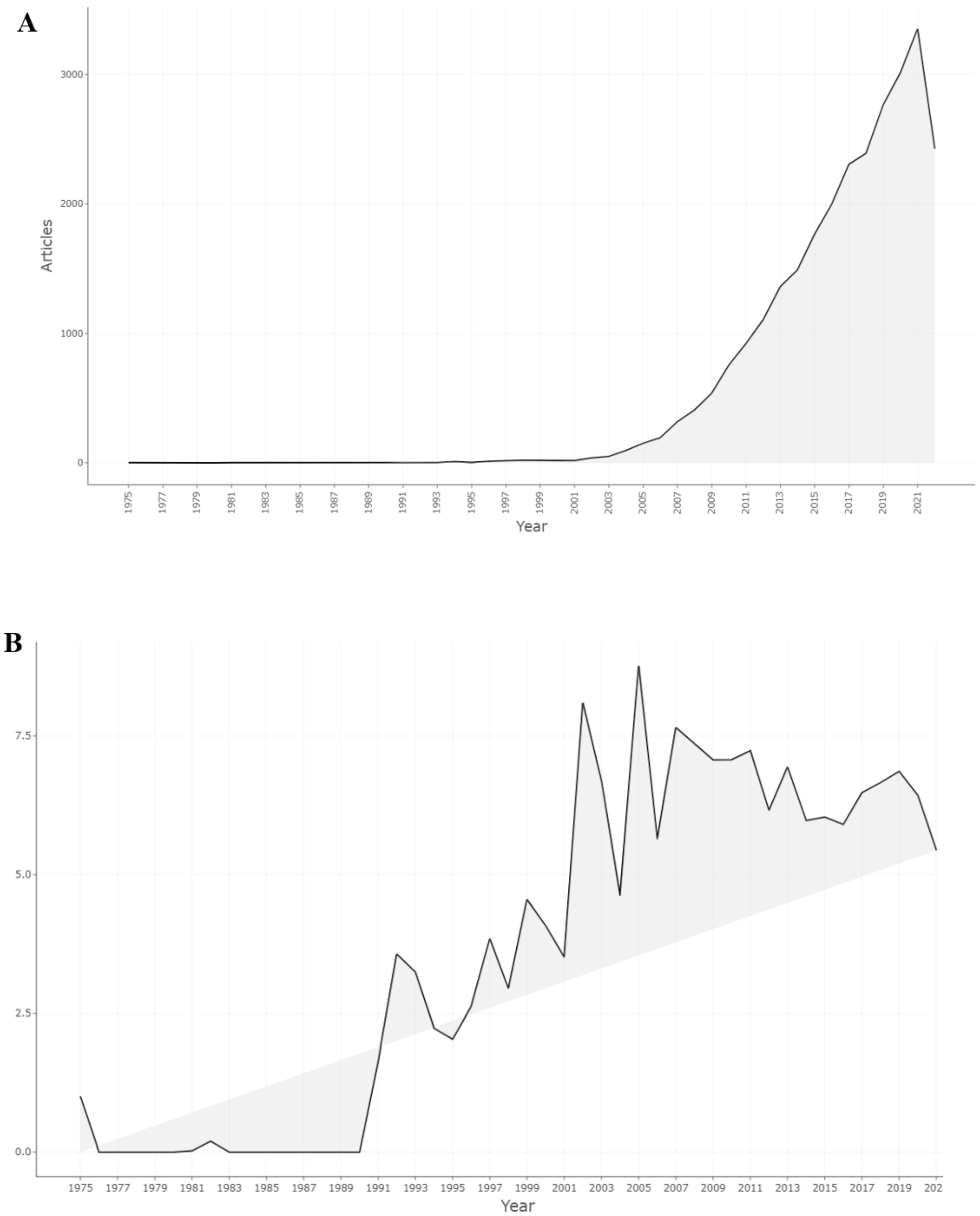

Supplementary Fig. S8. Annual scientific production (A) and average citation per year for references (B) (2000–2022).

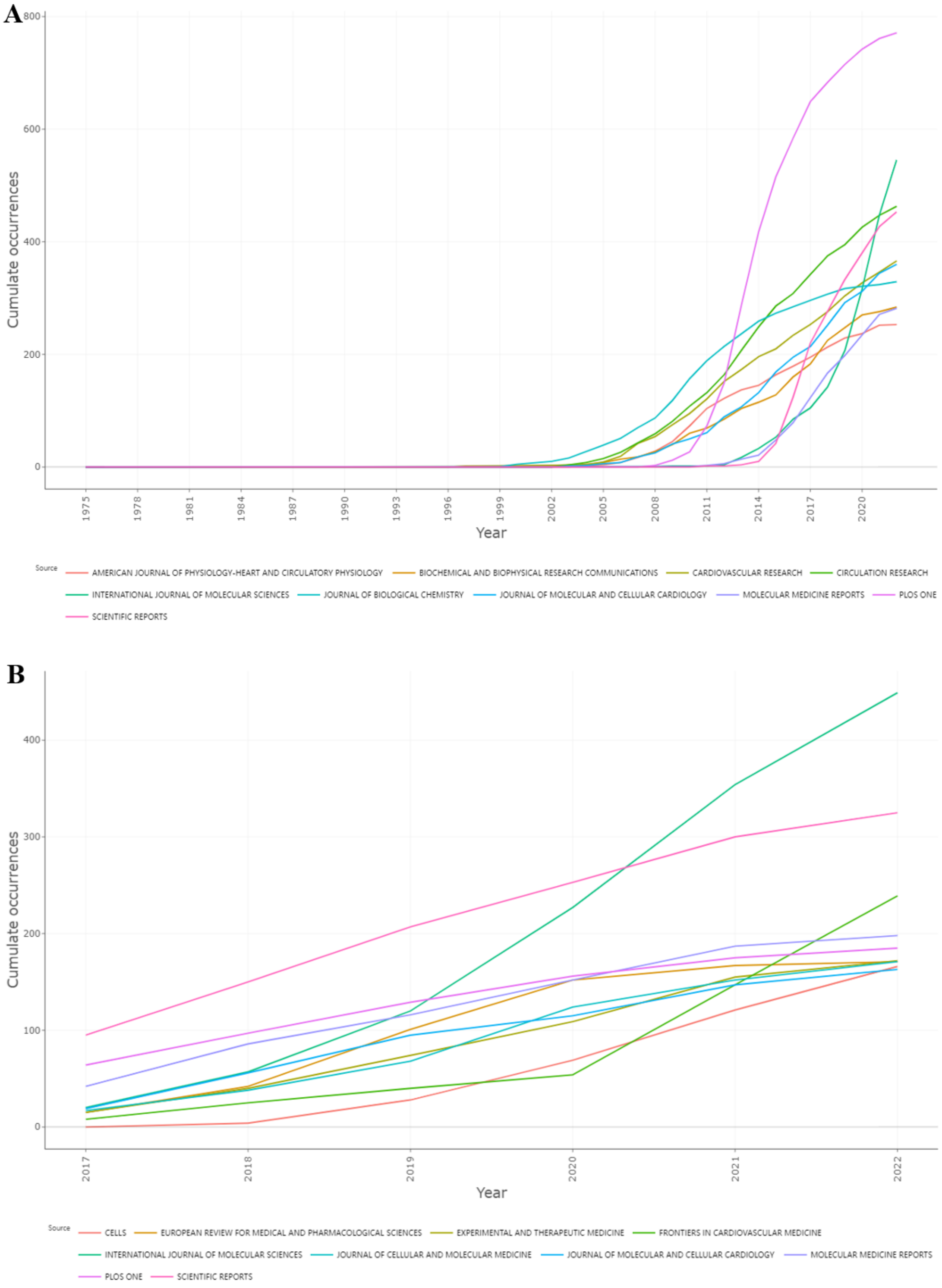

Supplementary Fig. S9. The top 10 growth sources of publications (2000–2022 and 2017–2022).

A

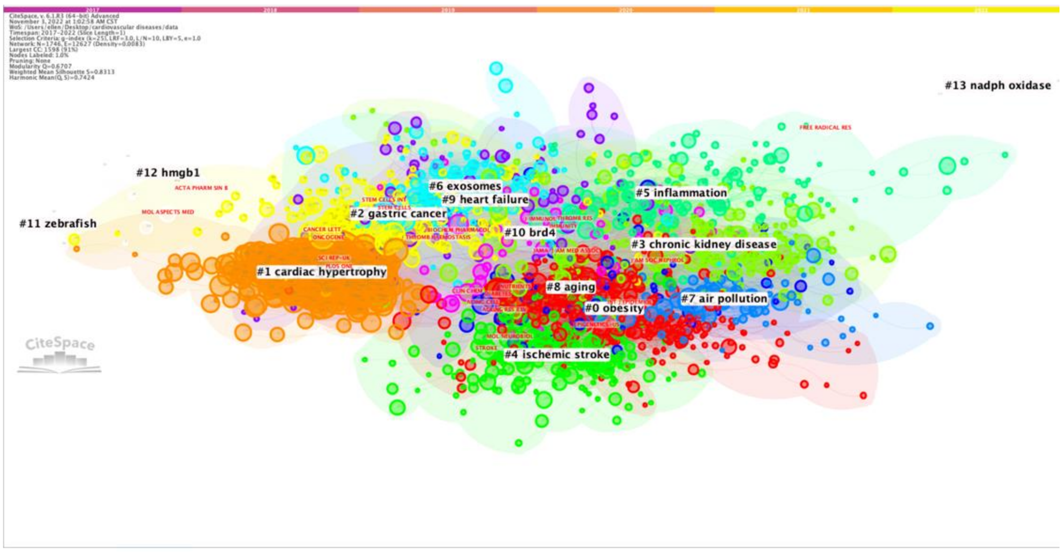

B

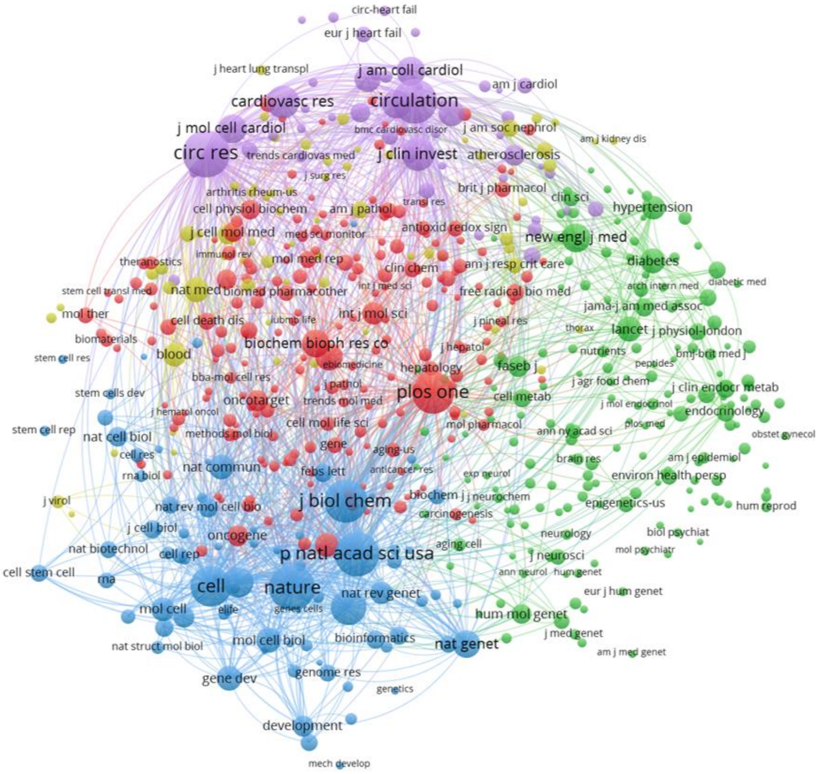

Supplementary Fig. S10. Overlay visualization of the most cited journals during the last five years (A), and the most co-cited journals that published the most articles during the last 20 years (B).

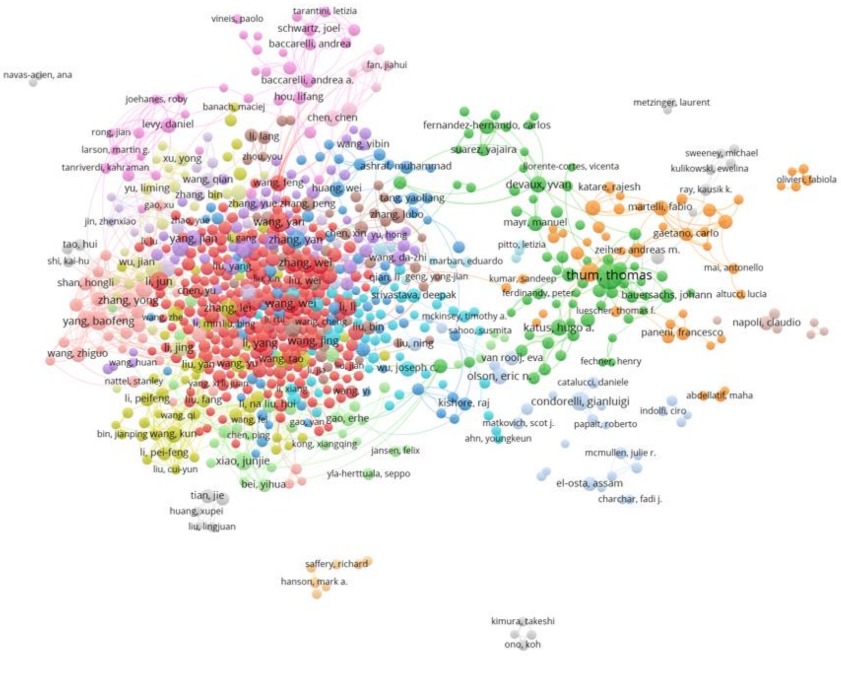

Supplementary Fig. S11. The co-authorship network obtained with VOSviewer.

A total of 24 clusters that comprise 875 different authors were identified. Minimum number of articles of an author should exceed 13.

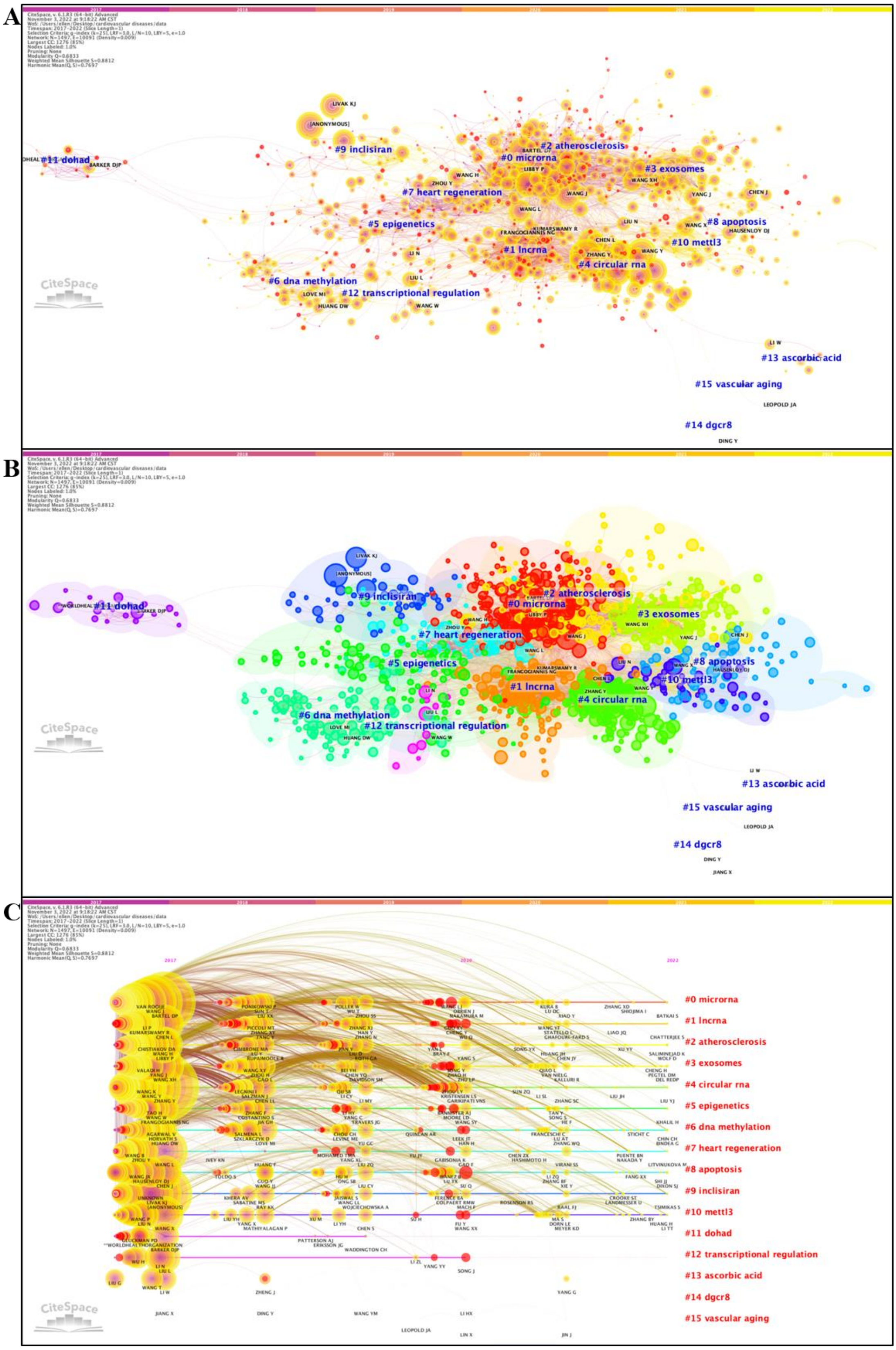

Supplementary Fig. S12. Co-citation network of authors (A), corresponding clusters (B), and timeline visualization of the network (C) for the time period 2017–2022.
